# All Roads Lead to Rome: Resting-State Criticality as a Neurophysiological Marker of Brain-Computer Interface (BCI) Performance in People with Amyotrophic Lateral Sclerosis (ALS) and Healthy Participants

**DOI:** 10.21203/rs.3.rs-10422321/v1

**Published:** 2026-09-29

**Authors:** Tomko Settgast, Oren Shriki, Georg Northoff, Federico Zilio, Seyyed Bahram Borgheai, Yalda Shahriari, Andrea Kübler

**Affiliations:** Julius-Maximilians-Universität Würzburg; Ben-Gurion University of the Negev; University of Ottawa; University of Padua; Emory University; University of Rhode Island; Julius-Maximilians-Universität Würzburg

**Keywords:** Amyotrophic Lateral Sclerosis, Brain Criticality, Brain-Computer Interface, Complete Locked-In State, Windows of Consciousness

## Abstract

This exploratory study investigates the relationship between resting-state brain dynamics (EEG) and subsequent brain-computer interface (BCI) performance in people with amyotrophic lateral sclerosis (ALS) and healthy participants. Grounded in the critical brain hypothesis, we examined whether neurophysiological markers of criticality in resting-state EEG correlate with subsequent BCI performance. We analyzed neuronal avalanche dynamics, multiscale criticality measures, the spectral slope, and Lempel-Ziv complexity across two datasets: a longitudinal single-case study of a person in the locked-in state after a disease history with ALS (Dataset 1) and longitudinal data from 5 people with ALS and single sessions from 6 healthy participants (Dataset 2). Neuronal avalanche dynamics, the spectral slope, and Lempel-Ziv complexity consistently correlated with subsequent BCI performance in people with ALS. Conversely, healthy participants showed inverted correlation patterns for the neuronal avalanche dynamics. While group comparisons were not statistically significant, effect size analyses indicated supercritical brain dynamics in people with ALS. We integrate these findings into the B-ARC (BCI-Aptitude through Resting-State Criticality) framework proposing an inverted U-shaped relationship between the proximity of resting-state brain activity to criticality and BCI control. In this framework, healthy participants approach the critical optimum from a subcritical regime, while people with ALS appear to approach it from a putatively supercritical regime characterized by cortical hyperexcitability. These findings and the proposed framework provide potential resting-state predictors of BCI aptitude in people with ALS and may inform the detection of so-called *Windows of Consciousness*.

## Introduction

*Amyotrophic lateral sclerosis* (ALS) is an incurable, progressive neurodegenerative disease (Zarei et al. 2015) presenting as a combination of upper and lower motor neuron dysfunction with multiple phenotypes (Feldman et al. 2022). Although cognitive impairment occurs, warranting its classification as a *multisystem disease* (Borgheai et al. 2025), ALS is primarily characterized by the continuous loss of motor control, leading to progressive paralysis that also affects speaking, swallowing, and breathing (Zarei et al. 2015; Feldman et al. 2022). While artificial ventilation can prevent premature death, it can also lead to classic or even complete locked-in state (LIS and CLIS, respectively) (Zarei et al. 2015; Kellmeyer et al. 2018), with CLIS being characterized by the total loss of voluntary movement and communication ability (Kübler 2020).

Brain-computer interfaces (BCI) aim to restore communication in CLIS by circumventing the need for muscular output (Kübler 2020). By definition, these assistive technologies measure and convert brain activity in (near) real-time into functionally useful outputs to replace, restore, enhance, supplement, or improve the natural outputs of the brain ([CSL STYLE ERROR: reference with no printed form.]). Most BCIs decode predefined electrical brain activity recorded either invasively (electrocorticography, ECoG; implanted single intracortical electrodes or electrode arrays) or noninvasively (electroencephalography, EEG) (Kübler 2020).

EEG signals commonly employed for BCI control include event-related potentials such as the P300, typically elicited within an oddball paradigm, steady-state evoked potentials elicited through repetitive visual, auditory, or tactile stimulation, and the sensorimotor or μ rhythm as well as slow cortical potentials elicited by specific imagery (Kübler 2020; Rashid et al. 2020). Initially presented by Farwell and Donchin (Farwell and Donchin 1988) using visual stimuli, P300-based BCIs were subsequently established across other modalities, that used, e.g., auditory and tactile stimulation (Allison et al. 2020). In these paradigms, a number of stimuli are presented and attention renders the target stimulus an oddball eliciting the P300 (Kübler and Birbaumer 2008; Kübler 2020).

EEG-based BCIs have successfully been applied for home-use in people in LIS in the past (Kübler 2020). Nevertheless, despite numerous attempts to establish BCI-based communication in CLIS (for a recent review, see Rezvani et al. (2023)), successful long-term use of a non-invasive EEG-based BCI has been rare^1^ – see, e.g., Okahara et al. (2018). Consequently, some authors have questioned previous claims of successful non-invasive BCI-based communication (Spüler 2019), or the feasibility of BCI-based communication for people in the complete locked-in state (pCLIS in the following) in general (Grosse-Wentrup 2019).

Kübler (2020) suggested that fluctuations in the state of consciousness might explain failures of BCI-based communication in CLIS. This challenges the assumption that consciousness remains unaffected in CLIS and suggests that targeting BCI use at so-called *Windows of Consciousness* (WoC) could prevent failed communication attempts. Recent studies have reported such fluctuations in neurophysiological markers of consciousness in CLIS (Zilio et al. 2023; Adama and Bogdan 2025) and some authors proposed that CLIS be reclassified as a *disorder of consciousness* (DOC) (Gobert et al. 2025). To effectively target BCI use in pCLIS during promising WoC, reliable resting-state predictors of BCI aptitude are required that can later be detected in spontaneous brain activity.

Although resting-state EEG has been used to predict BCI performance, previous studies have exclusively investigated healthy participants, with Borgheai et al. (2024) being the only exception. Furthermore, the studies have primarily either focused on trait differences (Halder et al. 2013), or non-P300-based BCI paradigms (Blankertz et al. 2010; Ahn et al. 2013; Zhang et al. 2013). This is surprising, as P300-based BCIs are considered convenient, reliable and user-friendly (Hammer et al. 2018; Won et al. 2019) and show higher effectiveness, efficiency and satisfaction than sensorimotor-based BCIs (Kübler et al. 2014).

Shin et al. (2020) investigated resting-state EEG predictors of P300-based BCI performance in healthy participants. They reported negative correlations between delta-frequency band power and subsequent BCI performance, as well as negative correlations with delta- and alpha-frequency band connectivity and a positive correlation with gamma-frequency band connectivity. More recently, Borgheai et al. (2024) demonstrated that BCI performance can be predicted from pre-screening measures. Using multivariate linear regression models, they found that temporo-parietal delta-frequency band power at rest emerged as a significant negative predictor for one of their visuo-mental BCI protocols. Notably, these models were trained on data from both healthy participants and people with ALS (pALS in the following). These findings implicate the potential of resting-state brain activity to predict subsequent BCI performance – a necessary pre-requisite to find WoC.

In an attempt to identify potential markers for WoC, Settgast et al. (2023) investigated associations between resting-state brain dynamics and subsequent BCI performance in a longitudinal dataset from a single person in the locked-in state after a disease history with ALS (pLIS-ALS in the following). They used two neurophysiological measures: *Lempel-Ziv complexity* (LZC) – reflecting complexity as the randomness of finite sequences such as discrete-time physiological signals (Aboy et al. 2006) – and the 1/f aperiodic component of the EEG power spectrum, hereafter referred to as *spectral slope*^2^. These measures reliably distinguish between different states of consciousness (Zilio et al. 2023; Medel et al. 2023; Höhn et al. 2024; Maschke et al. 2024b) and are altered in CLIS (Zilio et al. 2023).

LZC has also been investigated in relation to BCI control before. Wang et al. (2022) reported that lower alpha-band LZC at rest was related to better subsequent motor imagery BCI performance in healthy participants. More recently, Adama and Bogdan (2025) introduced a method for detecting episodes of consciousness in CLIS. This method correlated with performance with an electrooculogram(EOG)-based communication system in one person in CLIS, and LZC was identified as a key contributor to this measure of consciousness. This association likely stems from the fact that LZC is considered a measure of brain signal entropy (Agnorelli et al. 2025), which has been proposed to index the richness of conscious experience (Carhart-Harris 2018). Similarly important to BCI use, Bettencourt and Nunes (2024) demonstrated that the spectral slope (referred to as the power-law exponent, PLE) significantly differentiated between successful and unsuccessful BCI sessions in a person in CLIS.

Against this background, Settgast et al. (2023) and Settgast and Kübler (2024) examined the relationship between LZC and the spectral slope (PLE) in resting-state brain activity and subsequent BCI performance. Their findings revealed a striking discrepancy: A single pLIS-ALS exhibited a positive correlation between spectral slope steepening at rest and subsequent performance with a tactile P300-based BCI, alongside a negative correlation for LZC (Settgast et al. 2023). In contrast, healthy participants showed the inverse pattern: across participants higher LZC values and flatter slopes at rest were associated with better performance (visual P300-based BCI) (Settgast and Kübler 2024). To reconcile these conflicting results the authors proposed a non-linear relationship between brain dynamics and BCI performance, framed within the *critical brain hypothesis* (O’Byrne and Jerbi 2022) (see Fig. 1).

This hypothesis posits that the brain operates near a critical transition point between order and randomness/disorder (O’Byrne and Jerbi 2022; Algom and Shriki 2026) – an optimal state for information processing, due to higher sensitivity to stimuli and long-range integration (Shriki and Yellin 2016). Deviations toward subcritical and supercritical regimes are associated with reduced information-processing capacity and flexibility (O’Byrne and Jerbi 2022; Müller et al. 2025; Algom and Shriki 2026) – and, in more extreme deviations, to loss of consciousness (Algom and Shriki 2026).

Although the existence of a scale-free power spectrum, i.e., the power law scaling in the spectral domain indicated by the spectral slope, is, by itself, considered insufficient to indicate criticality (O’Byrne and Jerbi 2022; Ahmad et al. 2022), reported relationships between brain criticality and scale-free spectral properties quantified by the spectral slope (Wainio-Theberge et al. 2022; O’Byrne and Jerbi 2022; Ahmad et al. 2022), as well as between brain criticality and LZC (Fekete et al. 2018), support a criticality-based interpretation of the opposing patterns observed in the pLIS-ALS versus healthy participants. Specifically, brain activity closer to criticality has been associated with flatter spectral slopes (Pascarella et al. 2025) and increased complexity (Mago et al. 2025). Accordingly, in healthy participants, BCI performance appeared to increase as resting-state brain activity approached criticality (Settgast and Kübler 2024) (see Fig. 1), from a slightly subcritical regime with its more stable dynamics (Algom and Shriki 2026) – subcriticality being considered characteristic of healthy brain activity (Priesemann et al. 2014; O’Byrne and Jerbi 2022; Fuscà et al. 2023). Conversely, the conflicting observations for the single pLIS-ALS (Settgast et al. 2023) can be reconciled by the hypothesis that this person’s resting-state brain activity resided within a slightly supercritical regime. This aligns with established findings in the pathophysiology of ALS, i.e., the observed cortical hyperexcitability (Gunes et al. 2022; Angiolelli et al. 2026), a flatter spectral slope in resting-state magnetoencephalography (MEG) (Trubshaw et al. 2024), and the relationship between excessive excitation, e.g., due to neurodegeneration, and supercritical brain dynamics, e.g., observed in Alzheimer’s disease (Javed et al. 2025).

These putatively slightly supercritical dynamics in ALS allow for the reconciliation of the opposing findings for pALS and healthy participants. On the basis of the described observations and outlined considerations, we propose the *B-ARC* (BCI-Aptitude through Resting-State Criticality) *framework*: Under this framework, the relationships between resting-state brain criticality and BCI performance follow an inverted U-shape (see Fig. 1), where both groups show optimal performance as they approach the critical transition point at rest from opposite directions.

As a first step to reliably detect correlates of WoC, we here investigated the relationship between resting-state brain criticality and subsequent BCI performance in pALS. The present exploratory study sought to replicate and extend the previous findings reported in Settgast et al. (2023) by incorporating a broader selection of neurophysiological markers of criticality, including neuronal avalanche dynamics and multiscale criticality analyses (Fekete et al. 2018, 2021). Secondly, we applied these markers to a second independent dataset (Borgheai et al. 2024) to test the replicability of the findings in a larger group of pALS. Because Dataset 2 also included healthy participants undergoing the same experimental procedure, we further evaluated whether the previously observed pattern for healthy participants – namely, the approach toward criticality at rest with increasing BCI performance (Settgast and Kübler 2024) – could be replicated. Finally, we compared the criticality markers between healthy controls and pALS to identify systematic group differences, such as the flatter spectral slope recently reported for pALS (Trubshaw et al. 2024) and to evaluate the assumption of supercritical brain dynamics in ALS.

## Methods

### Participants and Datasets

#### Dataset 1: Longitudinal Investigation of BCI Use in a Single pLIS-ALS

Dataset 1 was recorded as part of a longitudinal study investigating long-term home use of a tactile P300-based BCI in a single person in the locked-in state after a disease history with ALS (pLIS-ALS) (Ziebell et al. 2024). The male person, 55 years old at the start of recording in 2018, was diagnosed with ALS in 2010 and had been in LIS since 2015 (ALSFRS-R score: 0). He requires invasive mechanical ventilation and artificial nutrition. Throughout the study he communicated via eye-blinks and eye-tracker.

The data represent a sub-selection of recordings from 41 home visits between October 2020 and May 2021. The final 10 of these visits included 1–2 minute, eyes-open (no fixation) resting-state EEG recordings prior to BCI use. These 10 sessions were used to investigate the correlation between resting-state EEG and BCI performance.

EEG data were recorded at 512 Hz with 12 active *g.LADYbird* Ag/AgCl electrodes attached to a *g.GAMMAcap* (Fz, FC1, FC2, C3, C1, Cz, C2, C4, Cp1, Cpz, Cp2, and Pz according to 10–10 system) using *BCI2000* and a *g.USBamp* amplifier (*gTec*, Austria). The ground electrode was positioned anterior to Fz and the reference electrode was attached to the right earlobe. During the recording the data were bandpass-filtered between 0.1 and 60 Hz, and notch-filtered between 48 and 52 Hz in real-time.

A 6-class tactile P300-based BCI was used. Tactile stimulation was delivered via vibrators attached to 6 locations on the person’s body. Each session consisted of at least 3 runs comprising 6 trials, i.e., 6 selections, per run. Each trial consisted of 8 stimulus sequences. Within a stimulus sequence, all vibrators were activated once in a randomly counterbalanced order. Each vibration lasted for 220 ms, followed by a 400 ms inter-stimulus interval (ISI). BCI performance was quantified as the reached online accuracy during a copy spelling task (Kübler et al. 2001). This task classically refers to copying words or letters that are presented to the user – here, selecting the right vibrators in the sequence presented to the pLIS-ALS. For our analysis, BCI performance was quantified as the mean BCI online accuracy and its coefficient of variation across the first three runs of a session.

#### Dataset 2: BCI Use in People with ALS and Healthy Participants

Dataset 2 was recorded as part of a larger study aiming at predicting BCI performance variability based on pre-screening (Borgheai et al. 2024). The data included 5 people with ALS (pALS; mean age: 57.71 years ± 6.8; mean ALSFRS-R score: 26.71 ± 15.36, min: 0, max: 42; mean disease duration: 6.3 ± 3.4 years; 2 females) and 6 healthy participants (mean age: 58.5 ± 5.55 years; 2 females). We analyzed the initial resting-state EEG recording (5 minutes, eyes open) and used the BCI performance achieved in the subsequent visual P300-based BCI copy spelling task.

Each person with ALS completed 3 to 5 sessions, with one session per day every 2 to 4 weeks. For our analysis, we excluded the so-called familiarization session of each person as procedural compliance could not be guaranteed. For healthy participants, only one session was included, as only that session followed the required order of a resting-state recording, preceding all other tasks.

EEG data were recorded at 256 Hz with 16 electrodes (Fz*, F5*, F6*, Fp1*, Fp2*, Cz, P3, Pz, P4, T7, T8, P7, P8, PO7, Po8, and Oz) using *BCI2000* and a *g.USBamp* amplifier (*gTec*, Austria). The ground electrode was positioned at Fcz and the reference electrode at the right earlobe. The asterisk (*) denotes the closest 10–10 system channel position, as the standard positions were partially occupied by fNIRS emitters/detectors for simultaneous fNIRS recording.

The BCI application used was a standard visual P300-based 6×6 letter matrix for spelling (Nijboer et al. 2008). During each trial, rows and columns were randomly intensified and the letters of the highlighted row/column were superimposed by the face of a celebrity (93.75 ms duration, followed by 62.5 ms ISI), a standard procedure to increase BCI performance (Kaufmann et al. 2013). For our analysis, the BCI performance reached during copy-pelling was used. This performance was quantified by the target accuracy that expresses the correctly classified epochs, referred to as *BCI_Default_Accuracy*.

### EEG Pre-Processing

EEG data were pre-processed using *EEGLAB* (Delorme and Makeig 2004) following recommendations of Miyakoshi [NO_PRINTED_FORM] and supplemented with procedures to address rank deficiencies prior to independent component analysis (ICA) to prevent the occurrence of so-called ghost independent components (Kim et al. 2023).

Firstly, Dataset 1 was resampled to 256 Hz to align with Dataset 2. We then applied, a high-pass filter at 1 Hz to ensure robust ICA decomposition (Winkler et al. 2015) and a low-pass filter at 40 Hz to exclude potential artifacts of the online notch filter in Dataset 1. We used the *clean_artifacts* function (EEGLAB) with GUI default parameters to detect and remove flatline (more than 5 seconds), abnormally correlated (minimally accepted correlation with nearby channels: 0.85) and noisy channels (more than 4 standard deviations of line noise relative to its signal). All other data cleaning procedures of this function were disabled at this stage. Removed channels were spherically interpolated. Data were then re-referenced to the *common average* using a rank-preserving method (adding a zero-filled dummy reference channel before re-referencing and removing it subsequently). The common average re-reference was chosen as empirical results indicate its superiority over monopolar references such as earlobe reference electrodes (as used in both datasets here), at any number of electrodes (Hu et al. 2018). This was followed by a second pass of the *clean_artifacts* function to detect and correct short time bursts and artifacts using artifact subspace reconstruction (GUI default parameters: maximally acceptable 0.5 second standard deviation = 20). All bad channel detection algorithms were disabled at this stage. EEG data were then decomposed into independent components using the *runica* function (EEGLAB). To prevent ghost components that occur due to interpolated channels, the effective rank was estimated before ICA by applying the following equation, as recommended in Kim et al. (2023): *dataRank* = *sum* (*eig*(*cov*(*double*(*EEG.data*′))) > 1*E* − 7). When rank deficiencies were detected, the estimated rank was used as an input parameter for the “pca” option of the *runica* function. Finally, artifact components were automatically detected and removed using *MARA* (Winkler et al. 2011).

### Feature Extraction

Following pre-processing, we extracted a set of neurophysiological markers of criticality from the resting-state EEG (see Table 1 for an overview). Due to the nature of the recordings, feature extraction was performed on EEG data of varying lengths. To improve consistency, data were truncated (see below).

#### Dataset 1

The resting-state EEG data were truncated to 95 s, starting 5 s after recording onset. Session 5 was an exception, as it was accidentally recorded for a substantially shorter time, leaving only 61.59 s after pre-processing. We truncated this session, starting 5 s after recording onset and cutting off roughly 1.5 s at the end, resulting in a length of 55 s.

#### Dataset 2

The feature extraction for Dataset 2 was equal for healthy participants and pALS. In accordance with the original publication, we analyzed a 4 minute epoch from the middle of the resting-state recording leaving out the first and last 30 s (Borgheai et al. 2024).

### Neurophysiological Markers of Criticality (EEG)

#### Neuronal Avalanche Analysis

Neuronal avalanches are spatiotemporal bouts of activity (Beggs and Plenz 2003; Shriki et al. 2013; Fekete et al. 2021). To identify them in multi-sensor array data, such as EEG, the continuous signals are first discretized by applying an amplitude threshold, after which data are divided into discrete time bins (Fekete et al. 2021). Any value exceeding the threshold is classified as an event (Shriki et al. 2013), and an avalanche is defined as a continuous sequence of time bins containing at least one event in one sensor (here EEG electrodes), ending with a time bin that contains no events in any sensor (Shriki et al. 2013; Fekete et al. 2021).

Neuronal avalanches are characterized by the distributions of their fundamental properties (i.e., size and duration (Fekete et al. 2021)), and by the *branching parameter* (σ, Sigma) (Shriki et al. 2013). The branching parameter quantifies the average ratio of the number of events in one time bin to the number of events in the previous time bin (typically, the first two bins of an avalanche are considered) (Shriki et al. 2013). At criticality, σ = 1, meaning that activity can travel long distances through the neural system without dying out or causing *runaway excitation* (Shriki et al. 2013). Values of σ > 1 or σ < 1 are considered indicative of supercritical or subcritical dynamics, respectively (O’Byrne and Jerbi 2022). However, measured branching parameters, e.g., in MEG and EEG, may vary slightly around 1 while the underlying neural dynamics can still be considered near-critical (Shriki et al. 2013; Meisel et al. 2013; Arviv et al. 2015). Neural systems at criticality also exhibit power law distributions for avalanche size and duration (Meisel et al. 2013; O’Byrne and Jerbi 2022). At criticality, these distributions obey power laws whose exponents have been theoretically predicted to be −3/2 for the avalanche size (denoted as alpha (α)), and − 2 for the avalanche duration (denoted as tau (τ) – also sometimes referred to as beta (β)) for a critical branching process (Arviv et al. 2015; Tian et al. 2022). Empirically observed exponents often deviate from these predicted values due to factors such as the experimental method, recording conditions, and inter-individual variability (Girardi-Schappo 2021).

### Parameter Selection for Neuronal Avalanche Analysis

The low number of electrodes in both datasets (Dataset 1: 12 channels, Dataset 2: 16 channels), and the uneven scalp coverage in Dataset 1 may have predisposed our analysis to subsampling effects for the neuronal avalanche estimation (Levina and Priesemann 2017). To mitigate this, we deviated from standard parameters used for high-density sensor arrays, such as Δt ≈ 4–20 ms, and a ± 3 SD threshold (Lombardi et al. 2021). Instead, we applied a data-driven approach to determine the optimal values for our time bin (Δt) and threshold. We systematically varied these parameters and selected the combination with the lowest value for the timescale and highest value for the threshold that yielded an expected strong positive correlation between the branching parameter, σ, and mean LZC (LZC_mean) – for a comparison, see e.g., Maschke et al. (2024b).

The expected positive correlation was confirmed for Dataset 1 (*ρ* = .70, *p* = .025; see Fig. 2, and Online Resource 1, Table S1) and for the pALS in Dataset 2 using Spearman’s rank correlation (*ρ* = .93, *p* < .001; see Online Resource 1, Figure S1, Table S2). This yielded optimal parameters of Δt = 10 samples (~ 39.1 ms at a sampling rate of 256 Hz) and a threshold of ± 2 SD. While a ± 2 SD threshold is more lenient than conventional values and potentially risks noise being misclassified as neural events (Shriki et al. 2013), we argue that the selected rigorous pre-processing strategy and our stringent data rejection criteria (see Sliding Time Window Analysis and Data Quality Control) should have effectively reduced the impact of noise. We acknowledge that we did not observe the expected correlation between σ and LZC_mean for the healthy participants in Dataset 2. Instead, they showed a non-significant anticorrelation (*ρ* = − .66, *p* = .156, see Fig. 5, Table S3).

We verified that the chosen parameter values produced roughly neurophysiologically plausible results for the healthy participants in Dataset 2. The mean values across the 6 participants were: σ = 0.96 (SD = 0.07), α = −0.99 (SD = 0.05), and τ = −1.78 (SD = 0.22). The observed deviations from the values for σ, α and τ at criticality, i.e., 1, −3/2 and − 2 respectively (Arviv et al. 2015), are likely due to subsampling effects (Levina and Priesemann 2017).

### Multiscale Criticality Analysis

To account for the timescale-dependent estimation of neuronal avalanches, we also calculated multiscale criticality features (Fekete et al. 2018, 2021) for σ and α. This method calculates the neuronal avalanche dynamics (σ & α) across a range of timescales and plots them against the used timescales. The resulting distributions follow a curve whose slope (A) and intercept (B) are informative for the underlying neural state (Fekete et al. 2021). We calculated the multiscale criticality features using timescales between 6 and 14 samples in steps of 2.

All the selected criticality markers related to neuronal avalanches, i.e., the neural branching parameter (σ), the power law exponents of the avalanche size distribution (α), and duration distribution (τ) as well as the multiscale criticality features, A, and B for σ, and α, were calculated across all channels on the entire length of the pre-processed EEG recordings. The calculations were performed using custom-made MATLAB scripts created by author TS, implementing functions generously provided by author OS.

## Absolute and Relative Delta Power

To approximate a process referred to as *critical slowing-down* describing an increase in power in ultra-slow frequencies of the power spectrum as networks approach criticality (Yellin et al. 2025), we calculated the absolute and relative power of the delta-frequency band (1–4 Hz). These metrics were calculated for each channel across the entire length of the pre-processed recordings.

We estimated the power spectrum for each channel of each recording using *pwelch* on the whole length of the recording by means of the *spectopo* function of the EEGLAB toolbox, returning a logarithmic PSD (dB). Then, we converted the logarithmic PSD to a linear power scale (μV^2^/Hz) to enable power integration. *Absolute delta power* was calculated by integrating the linear power spectrum between 1 and 4 Hz using a trapezoidal function (*trapz*). To calculate the *relative delta power*, we first obtained total power across the entire analyzed spectrum (1–40 Hz; *trapz*), then divided absolute delta power by absolute power across the entire 1–40 Hz spectrum. The channel-wise values were averaged across channels and used for further statistical analysis.

The calculations were performed using a custom-made MATLAB script created by author TS.

## Sliding Time Window Analysis and Data Quality Control

To investigate the temporal dynamics of criticality, we calculated a subset of criticality markers referred to as criticality-related features (Maschke et al. 2024b) in sliding time windows. These features include the exponents of the spectral slope (power-law exponent (PLE) and the exponent of the aperiodic (1/f) PSD component from the FOOOF toolbox, referred to as spSlopeExp; see below for details) and LZC. This method is similar to that used in previous publications (Settgast et al. 2023; Zilio et al. 2023; Settgast and Kübler 2024).

An initial explorative analysis revealed that several time windows yielded unphysiological positive spectral slopes – i.e., power spectra in which power increased with frequency. We assume these slopes originated from high-frequency noise (e.g., muscle artifacts) within the relatively short time windows used for the sliding-window analysis, which probably led to issues with the power spectrum estimation. To address this, we implemented a three-level quality control procedure:

Firstly, we optimized the window length to improve the stability of the power spectrum estimation. Through systematic testing, we found that a 9 s window with 50% overlap minimized the total percentage of noisy data across datasets. Secondly, we excluded any 9 s window exhibiting a positive trend in the power spectrum in at least one channel (indicated by a negative value for PLE, or spSlopeExp) from further analysis (i.e., all values for each criticality marker calculated within this window, being PLE, spSlopeExp, spSlopeOffs, and LZC, were set to NaN). All other criticality markers were untouched by this procedure and still calculated upon the whole length of the datasets. This procedure resulted in final data losses of 4% in Dataset 1 and 28.4% for pALS and 0% for healthy participants in Dataset 2 (see Online Resources 2, 3, and 4).

Finally, to increase the robustness of our findings and avoid the influence of noisy data, we excluded any recording from further analysis if more than 50% of its time windows were contaminated with noise. This criterion resulted in the exclusion of three recordings in Dataset 2 (two recordings from person ALS-15, and one from person ALS-16) resulting in a final set of 13 resting-state EEG recordings for the pALS in Dataset 2. No exclusions were performed for Dataset 1 or the healthy participants in Dataset 2. After excluding recordings, the percentage of rejected time windows in the remaining recordings decreased to 12.8% for the resting-state recordings of pALS in Dataset 2.

## Feature Analysis in Sliding Time Window

The criticality-related features selected – Lempel-Ziv complexity (LZC), power-law exponent (PLE), and the parameters for the aperiodic (1/f) PSD component from the FOOOF toolbox (i.e., exponent and offset; see below) – were calculated individually for each channel in each 9 s time window. This process yielded time series for each criticality-related feature and each channel. We then calculated the mean and coefficient of variation (CV) values for each channel from these time series. Time windows flagged as contaminated with noise (see Sliding Time Window Analysis and Data Quality Control) were excluded. The channel-wise values (mean, CV) were averaged across channels and used for statistical analysis.

Automated exclusion of contaminated time windows and grand averaging were performed using custom-made MATLAB scripts provided by author GN’s group with adaptations made by the authors FZ and TS.

### Spectral Slope

We characterized the spectral slope, which reflects the aperiodic (1/f) component of the EEG power spectrum, using two algorithmic approximations: the power-law exponent (PLE) and the exponent and offset of the spectral slope from the FOOOF toolbox (Donoghue et al. 2020b). Although the PLE has been used in (C)LIS-ALS research before (Settgast et al. 2023; Zilio et al. 2023; Bettencourt and Nunes 2024), it does not control for confounding (Donoghue et al. 2020a; Gyurkovics et al. 2022) true periodic, i.e., oscillating, neural brain activity. To account for this, we also used FOOOF.

For both methods, we estimated the PSD (1–40 Hz) for each 9 s time window of each channel using a multitaper method (*mtspectrumc* function) with a frequency resolution of 2 Hz (tapers: [9 17], no padding).

## Power-law exponent

The power-law exponent (PLE) was calculated as described in Zilio et al. (2023). We logarithmically transformed the PSD in both frequency and power domains. Then, we applied a linear least squares regression (*polyfit* function) to the log-log transformed PSD. The PLE is the absolute value of the slope of this regression.

The complete computation of the PLE was made using a MATLAB-based implementation of an algorithm developed by author GN’s group with custom-made adaptations from author TS. The script was made publicly available (Northoff).

## Exponent and Offset of the Aperiodic (1/f) PSD Component Using FOOOF

The FOOOF (*fitting oscillations & one over f*) toolbox parameterizes a PSD by modeling the aperiodic component of neural spectra, as well as its overlying periodic peaks (Donoghue et al. 2020b). The aperiodic component is expressed by its exponent and offset.

We applied the FOOOF algorithm to the multitaper PSDs (1–40 Hz) calculated for each 9 s window of each channel. We used the MATLAB wrapper for the FOOOF toolbox (*FOOOF_mat* function) to perform the calculation. To fit the aperiodic component, we used the *fixed* aperiodic mode without a knee. This is consistent with other research conducted within this frequency range (1–40 Hz) – for example, see Wiesmann et al. (2024). For the periodic components, we chose a minimum peak width of 4 Hz (twice the frequency resolution) and a maximum peak width of 8 Hz. We kept all other settings to control the peak fits in the model at their default values. From the created FOOOF object, we extracted the offset and exponent of the aperiodic component (to which we refer as spSlopeExp and spSlopeOffs).

Any 9 s time window containing a negative exponent from either the power-law exponent (PLE) or the spSlopExp was considered contaminated by high-frequency noise and flagged for exclusion (see Sliding Time Window Analysis and Data Quality Control).

The used time-windows-based calculation of the aperiodic component was made with a custom-made MATLAB script created by author TS. The script was made publicly available (Northoff) after consultation with FOOOF (Donoghue et al. 2020b) developer, Thomas Donoghue (personal communication).

### Lempel-Ziv Complexity

When applied to EEG data, LZC reflects the regularity of the EEG signal (Zilio et al. 2023) as a function of pattern richness (Mago et al. 2025). LZC was calculated using the procedure described by Zilio et al. (2023). This procedure uses the computational method proposed by Zhang and Roy (2001) with the median as the binarization threshold due to its robustness to outliers (Nagarajan 2002). This is followed by the complexity calculation and a normalization to reflect the number of emerging patterns (Aboy et al. 2006).

According to Maschke et al. (2024a), observed changes in LZC may be partially attributed to variations in the spectral slope (see also Zilio et al. (2023), for a detailed analysis of this relationship). This relationship likely explains the consistently high negative correlations observed between these measures (Settgast et al. 2023; Settgast and Kübler 2024). Given these strong correlations, we assumed that LZC values would be similarly confounded by the high-frequency noise that affected the estimation of the exponents of the spectral slope (PLE, spSlopeExp). Therefore, LZC values were excluded from further analyses for any 9 s time window which has been flagged as contaminated (see Sliding Time Window Analysis and Data Quality Control).

LZC computation was made using a MATLAB script provided by author GN’s group. It is largely equivalent to an implementation made publicly accessible (Temporo-spatial 2022).

## Statistical Analysis

All statistical analyses were performed using RStudio (Version: 2025.09.2). Following recommended practices for exploratory research, we enhanced the robustness of our findings via a two-fold strategy (Szucs and Ioannidis 2017): 1) we performed our exploratory analyses on two independent datasets and 2) we focused on the magnitude and uncertainty of our findings, instead of on p-values alone, and report effect sizes along with confidence intervals. Consistent with this exploratory framework, no corrections for multiple testing were applied (Bender and Lange 2001). Since it is not possible to reliably test for normality in small samples such as the ones investigated (Le Boedec 2016), we followed recommendations to use the nonparametric Spearman’s rank correlation coefficient to test correlations (de Winter et al. 2016), and the Wilcoxon rank-sum test for group comparison which is to be preferred when population characteristics are unknown (Bridge and Sawilowsky 1999).

### Dataset 1

#### Resting-State EEG vs. BCI Performance (pLIS-ALS)

We investigated Spearman’s rank correlation coefficients between the resting-state neurophysiological markers of criticality and the subsequently achieved mean BCI performance (BCI_Online_Accuracy_mean) and its variability (BCI_Online_Accuracy_CV) across the 10 available sessions with resting-state EEG recordings.

### Dataset 2

#### Resting-State EEG Data vs. BCI Performance (pALS)

The primary objective of Dataset 2 was to determine whether the correlations between the resting-state neurophysiological markers of criticality and BCI performance observed for the single pLIS-ALS of Dataset 1 would exhibit comparable patterns in a larger pALS sample. After the exclusion of 3 sessions due to poor data quality (see Sliding Time Window Analysis and Data Quality Control), the analysis was conducted in the final set of 13 sessions.

To investigate if the associations between the criticality markers and BCI performance could be observed for each person with ALS, we used repeated measures correlations (rmCorr) (Bakdash and Marusich 2017). This method accounts for nonindependence of observations by estimating the common within-individual association between two variables while allowing for varying intercepts across subjects.

Prior to analysis, we visually inspected the subject-specific regression slopes (see spaghetti plots in Online Resource 1, Figure S2) to ensure consistent directionality of the within-subject associations. We then calculated rmCorr (r_rm_) for each criticality marker that met this criterion. Subsequently, rmCorr model assumptions were assessed using diagnostic plots (Q-Q plots, residuals vs. fitted/leverage) to ensure no severe violations were present (not shown).

Person ALS-11 was identified as an outlier for two criticality markers. For the mean power-law exponent (PLE_mean), this person exhibited insufficient within-subject variance (range: 1.4385–1.4389), resulting in an undefined regression slope (see Online Resource 1, Figure S3B). Consequently, ALS-11 was excluded from the analysis of this marker. This person also displayed an extreme value for absolute delta power (AbsDeltaPwr) as can be seen in Online Resource 1 (Figure S3A). The outlier behavior was confirmed by diagnostic plots identifying a datapoint with high leverage corresponding to this extreme value, as well as by median absolute deviation (MAD) outlier analysis (threshold = 3). Accordingly, ALS-11 was also excluded from the rmCorr analysis for absolute delta power.

Notably, while the MAD outlier analysis also flagged mean Lempel-Ziv complexity (LZC_mean) of one data point as a potential outlier for ALS-11, diagnostic plots indicated that this data point did not exert undue leverage. Moreover, visual inspection of the diagnostic Q-Q plot did not reveal severe deviations from the normality assumption of residuals. To preserve as many data points as possible, and thus, biological variability, this participant was retained for all other variables.

Additionally, to investigate the interdependency of the criticality markers, we calculated pooled Spearman’s rank correlations across all sessions (N = 13 sessions from N = 5 pALS). We acknowledge that this approach treats repeated measurements from the same individuals as independent observations, potentially inflating statistical significance (Bland and Altman 1994). However, due to the small sample size, we chose this method to illustrate the consistency of criticality marker inter-correlations. We emphasize that this method was not used to answer our research question regarding the existence of associations between resting-state neurophysiological markers of criticality and subsequent BCI performance.

#### Resting-State EEG Data vs. BCI Performance (Healthy Participants)

We investigated Spearman’s rank correlation coefficients between the resting-state neurophysiological markers of criticality and the subsequent BCI performance across subjects.

#### Group Comparison (pALS vs. Healthy Participants)

To support our assumption that the relationship between the changes in the criticality-related features (LZC, PLE) and subsequent BCI performance observed for a pLIS-ALS (Settgast et al. 2023) reflects a shift away from *supercritical* brain dynamics, we exploratorily compared the resting-state neurophysiological criticality markers between the pALS (n = 5) and healthy participants (n = 6) of Dataset 2. Since the pALS underwent multiple recording sessions, only the first usable session for each person was included. For the majority, this corresponded to the first session after the familiarization session. However, as this first session was excluded for person ALS-16 due to poor data quality (see Sliding Time Window Analysis and Data Quality Control), the second session was used for this person. Given that the number of criticality markers exceeded the sample sizes, multivariate approaches were not feasible. Accordingly, we performed independent statistical tests for each variable using the nonparametric Wilcoxon rank-sum test. We report the *U*-values, the exact *p*-values, the *Hodges-Lehmann estimates* with their 95% confidence intervals, and effect sizes (rank-biserial correlation, r_rb_). Despite the visually apparent heterogeneity in some criticality markers, particularly within the ALS group, we refrained from excluding potential outliers given the small sample sizes to preserve biologically valid variance.

We acknowledge that differences in datasets (i.e., number of participants, number of recordings, and type of BCI) prevent direct quantitative comparisons between them. We emphasize that our goal was to perform a conceptual replication, investigating the relationship between resting-state brain activity – quantified via neurophysiological criticality markers – and BCI performance, as well as assessing the stability of the findings across individual pALS and BCIs.

## Results

### Dataset 1

#### Correlates of BCI Performance in Resting-State EEG (pLIS-ALS)

For the single person in the locked-in state (LIS) after a disease history with ALS (pLIS-ALS) in Dataset 1, we observed several strong correlations between resting-state neurophysiological markers of criticality and subsequent BCI performance (see Fig. 2 and Table 2). The findings revealed that higher mean BCI performance was associated with lower neuronal avalanche branching parameters and more negative size-distribution exponents, as well as reduced complexity, but with higher spectral slope values and increased delta power.

Specifically, mean BCI performance (BCI_Online_Accuracy_mean) correlated negatively with the neural branching parameter (σ (Sigma); *ρ* = − .69, *p* = .028) and the exponent of the avalanche size distribution (α (Alpha); *ρ* = − .76, *p* = .011). The latter indicates that a steeper avalanche size distribution – implying a lower probability of large neuronal avalanches at rest – is associated with better subsequent BCI performance. In parallel, we found a negative correlation between mean BCI performance and mean Lempel-Ziv complexity (LZC_mean; *ρ* = − .82, *p*= .003), suggesting increasing BCI performance with decreasing complexity at rest.

Conversely, positive correlations were observed between mean BCI performance and the mean spectral slope exponents (PLE_mean; *ρ* = .84, *p* = .002; spSlopeExp_mean; *ρ* = .84, *p* = .002). These results indicate that BCI performance improves as the spectral slope becomes steeper (i.e., higher absolute exponent values).

Additionally, we observed positive correlations between mean BCI performance and both relative (*ρ* = .74, *p* = .015) and absolute delta power (*ρ* = .71, *p* = .022), as well as with the mean offset of the aperiodic (1/f) component of the EEG power spectrum (spSlopeOffs_mean; *ρ* = .71, *p* = .021). Finally, mean BCI performance was negatively correlated with the mean coefficients of variation for the spectral slopes (PLE_CV: *ρ* = − .84, *p* = .002; spSlopeExp_CV: *ρ* = − .80, *p* = .006) and the intercept of α as a function of timescales (MSCr_Alpha_B; *ρ* = − .76, *p* = .010).

The negative correlation between mean BCI performance and the exponent of the avalanche size distribution (α) was also visually evident (Fig. 3). The avalanche size distribution for the session with the highest mean BCI performance (83%; red) exhibited a substantially steeper slope (α = −0.96), i.e., a lower probability for larger neuronal avalanches, as compared to the session with the lowest mean BCI performance (50%; α = −0.68; see Fig. 3, blue). While both values deviate from the expected exponent of neural activity at criticality (α = −3/2), the exponent from the high-performance session was substantially closer to the expected value. Due to the small number of electrodes in the dataset, we did not observe large avalanches.

### Dataset 2

#### Correlates of BCI Performance in Resting-State EEG

##### People with ALS

An initial visual inspection (spaghetti plots) revealed substantial heterogeneity in the relationships between resting-state neurophysiological markers of criticality and subsequent BCI performance for the people with ALS (pALS) of Dataset 2. Despite this inter-individual variability, consistent directionalities for within-subject associations between BCI performance and several resting-state criticality markers were evident: the exponent of the avalanche size distribution (α), absolute delta power, mean Lempel-Ziv complexity (LZC_mean), the mean exponents of the spectral slope (PLE_mean, spSlopeExp_mean), and the mean offset of the aperiodic (1/f) component of the EEG power spectrum (spSlopeOffs_mean) (see Online Resource 1, Figure S2). Repeated measures correlations (rmCorr) were conducted to statistically investigate these common within-individual relationships.

rmCorr analyses partially replicated the patterns from Dataset 1 (Fig. 4): higher BCI performance was associated with more negative neuronal avalanche size-distribution exponents and lower complexity, but higher values in the spectral slope and delta power.

Specifically, BCI performance (BCI_Default_Accuracy) correlated negatively with the exponent of the avalanche size distribution (α (Alpha); *r*_*rm*_ (7) = − .83; *p* = .005). This suggests that a steeper distribution at rest – and therefore a lower probability for large neuronal avalanches – was associated with better subsequent BCI performance. In parallel, we observed increasing BCI performance with decreasing mean Lempel-Ziv complexity at rest (LZC_mean; *r*_*rm*_ (7) = − .89, *p* = .001).

In contrast, we found a positive correlation between BCI performance and the mean exponent of the aperiodic (1/f) component of the EEG power spectrum suggesting a positive association between the steepening of the spectral slope and BCI performance (spSlopeExp_mean; *r*_*rm*_ (7) = .69, *p* = .041). A similar positive association was found for the mean power-law exponent (PLE_mean; *r*_*rm*_ (6) = .74; *p* = .034) after excluding person ALS-11 due to their influential outlier behavior (see Statistical Analysis, Dataset 2). Additionally, we observed a common within-individual association between BCI performance and absolute delta power after the exclusion of person ALS-11 (AbsDeltaPwr; *r*_*rm*_ (6) = .8; *p* = .016).

The common-within individual association between the mean offset of the aperiodic (1/f) component of the EEG power spectrum and subsequent BCI performance only approached statistical significance (spSlopeOffs_mean; *r*_*rm*_ (7) = .6; *p* = .087).

## Healthy Participants

We found significant correlations between resting-state neurophysiological markers of criticality and subsequent BCI performance in the healthy participants of Dataset 2 (N = 6) (Fig. 5, Table 3). Crucially, one of the criticality markers exhibited an inverted pattern in healthy participants as compared to the pALS of Dataset 1 and 2: Unlike the pALS, higher BCI performance in the healthy participants was associated with a flatter avalanche size distribution.

In detail, we observed a perfect monotonic relationship between the exponent of the avalanche size distribution (α) at rest and subsequent BCI performance (BCI_Default_Accuracy) (α (Alpha); *ρ* = 1, *p* < .001). However, this result should be interpreted with extreme caution due to the very small sample size. Although a positive linear trend is visually apparent (Fig. 6), the magnitude of the observed effect is likely inflated. Nevertheless, statistically this trend indicates that a flatter avalanche size distribution at rest – i.e., a less negative α – is associated with better subsequent BCI performance in the healthy participants group.

This pattern is corroborated by a similarly strong, positive correlation for the exponent of the avalanche duration distribution (τ (Tau); *ρ* = .83, *p* = .042). This correlation indicates that a flatter avalanche duration distribution at rest – i.e., an increased probability of longer-lasting neuronal avalanches – is also associated with better subsequent BCI performance in healthy participants.

Additionally, healthy participants exhibited a negative correlation between BCI performance and the mean coefficient of variation for Lempel-Ziv complexity (LZC_CV; *ρ* = − .94, *p* = .005) and a positive correlation with the mean offset of the aperiodic (1/f) component of the EEG power spectrum (spSlopeOffs_mean, *ρ* = .83, *p* = .042).

### Group Comparison of Criticality Markers (EEG)

To investigate group differences in resting-state brain dynamics, we further conducted exploratory comparisons of resting-state neurophysiological markers of criticality between pALS (N = 5) and healthy participants (N = 6) from Dataset 2. For the pALS, only the first usable session was included to obtain a single datapoint per individual. Group comparisons were conducted independently for each criticality marker, using nonparametric Wilcoxon rank-sum tests.

While group comparisons did not reach statistical significance – likely due to limited power in this small sample – large and medium effect sizes indicated notable trends (see Table 4 for details) which are underpinned by visual differences in the distribution of these criticality markers (see Fig. 7).

A large effect size (*r*_*rb*_ = .53) suggested a steeper avalanche duration distribution (τ) for healthy participants (*Mdn* = −1.78, *IQR* = .33) than for pALS (*Mdn* = −1.59, *IQR* = .19). However, this difference did not reach statistical significance (*U* = 23, *p* = .177).

Regarding the neural branching parameter (σ), a medium-sized effect (*r*_*rb*_ = .4) indicated higher values in the ALS group (*Mdn* = 1.05, *IQR* = .07) than in healthy participants (*Mdn* = .98, *IQR* = .11). The Wilcoxon rank-sum test, however, revealed no statistically significant difference (*U* = 21, *p* = .329). Similar medium-sized effects were observed for the avalanche size distribution and the mean exponent of the aperiodic (1/f) component of the EEG power spectrum. Regarding the avalanche size (α), the effect size (*r*_*rb*_ = .4) pointed to a steeper distribution for healthy participants (*Mdn* = − .99, *IQR* = .08) than for pALS (*Mdn* = − .86, *IQR* = .18; *U* = 21, *p* = .329). The medium effect size (*r*_*rb*_ = .4) for the mean exponents of the aperiodic (1/f) component of the EEG power spectrum (spSlopExp_mean) pointed toward lower mean exponents in pALS (*Mdn* = .91, *IQR* = .31) than in healthy participants (*Mdn* = 1, *IQR* = .12; *U* = 9, *p* = .329) suggesting a flatter spectral slope in pALS. Neither of these trends reached statistical significance.

### Summary of Findings and Theoretical Alignment

To integrate the findings, Fig. 8 provides an overview of the relationships between the neurophysiological criticality markers at rest and subsequent BCI performance across both datasets and groups. This synthesis highlights the consistency of the correlations observed for the person in the locked-in state after a disease history with ALS of Dataset 1 and the pALS group of Dataset 2. Furthermore, for a subset of criticality markers – most prominently the variables referring to the neuronal avalanche dynamics, σ, α, and τ – healthy participants exhibited inverted correlations. Lastly, the overview illustrates that although group comparisons did not reach statistical significance, a large subset of criticality markers indicated a trend toward supercriticality in the pALS group of Dataset 2. A color code indicates alignment with the *B-ARC framework* (Fig. 1; discussed below) and the assumption for supercritical brain dynamics in ALS.

## Discussion

The main goal of this exploratory analysis was to identify in resting-state EEG recordings correlates of subsequent brain-computer interface (BCI) performance in people with amyotrophic lateral sclerosis (ALS; pALS in the following) and healthy participants. Identifying these correlates could eventually help to derive resting-state BCI aptitude predictors for these people. The proposed explanatory *B-ARC* (BCI-Aptitude through Resting-State Criticality) *framework* may also help inferring Windows of Consciousness (WoC) (Kübler 2020) for people in the complete locked-in state (CLIS; pCLIS in the following) in the future.

Neurophysiological markers of criticality may offer the opportunity to track such real-time changes in consciousness suggested by the WoC hypothesis. In their recently proposed ConCrit framework, Algom and Shriki (2026) outline how criticality supports consciousness, proposing that significant deviations into sub- and supercritical regimes result in the loss of consciousness. Therefore, monitoring changes in criticality may prove useful for indicating changes in conscious awareness in CLIS, thereby facilitating the detection of WoC.

Across two independently recorded datasets, we observed consistent correlations between resting-state neurophysiological markers of criticality and subsequent BCI performance in pALS. Most notably, the exponent of the neuronal avalanche size distribution (α) at rest showed a negative correlation with subsequent BCI performance (see Fig. 8). This pattern was evident in 10 sessions of a single person in the locked-in (LIS) state after a disease history with ALS (pLIS-ALS in the following; see Fig. 2 and Table 2) and replicated in a group of 5 pALS (see Fig. 4). Regardless of the disease stage or BCI modality, these findings suggest that a steeper avalanche size distribution at rest – i.e., a lower probability of large neuronal avalanches – is associated with better BCI performance in pALS.

Although the exact behavior of the power law exponents for the neuronal avalanche size and duration distribution (α, τ) in subcritical and supercritical regimes is not entirely clear, we posit that these exponents will decrease (i.e., become less negative) in supercritical states. This is because an increased probability of larger and longer-lasting neuronal avalanches leads to flatter size and duration distributions. We acknowledge that distributions in extreme cases of sub- and supercritical behavior no longer follow the power law, but exponential or bimodal distributions (Heiney et al. 2021) and may even be accompanied by a steeper slope at the initial part of the avalanche size distribution (Plenz et al. 2021). However, our framework focuses on subtle shifts to sub- and supercritical states where subjects are still conscious and power law approximations likely remain informative. This assumption of flatter distributions in supercritical states is supported by empirical evidence. Poil et al. (2012) demonstrated this for the size distribution of modelled network activity, and Fekete et al. (2018) reported smaller α and τ values in a disinhibited (i.e., supercritical) anesthetized rat barrel cortex. More recently, Harel et al. (2025) reported increasing σ values along flatter avalanche size distributions (α) as sleep deprivation accumulated in human participants – a state earlier interpreted as being supercritical (Meisel et al. 2013).

Accordingly, if the probability of larger and longer-lasting neuronal avalanches decreases as the system approaches critical dynamics from a supercritical regime, as indicated by steeper power law distributions (i.e., more negative exponents), then our findings suggest that pALS exhibited better BCI performance when their preceding resting-state activity shifted from supercritical dynamics towards critical dynamics. This interpretation is supported by the strong correlations between α and the neural branching parameter (σ) in both datasets (see Fig. 2 and Table S1; Figure S1 and Table S2).

Further support stems from so-called criticality-related EEG features (Maschke et al. 2024b), specifically the exponents of the spectral slope – represented by the power-law exponent (PLE) as well as the exponent of the aperiodic (1/f) component of the EEG power spectrum extracted using FOOOF (Donoghue et al. 2020b) (spSlopeExp) – and Lempel-Ziv complexity. Consistent with previous literature (Maschke et al. 2024b), we observed strong correlations between these measures and the neural branching parameter σ. Specifically, within the ALS cohort, we found that larger values of the neural branching parameter (higher σ) were associated with higher complexity (higher mean LZC values) and flatter spectral slopes (lower values for mean PLE and mean spSlopeExp).

While the exact physiological mechanism linking the spectral slope and criticality is still being investigated^3^, the spectral slope has repeatedly been shown to reflect changes in the excitation-inhibition (E:I) ratio (Gao et al. 2017; Nanda et al. 2023; Medel et al. 2023; Sheldon et al. 2025; Cortes-Briones et al. 2025). Specifically, the spectral slope has been discussed to reflect the combination of excitatory (glutamate AMPA receptors) and inhibitory (GABA receptors) synaptic currents (Gao et al. 2017; Medel et al. 2023), a view empirically supported by studies using GABAergic anesthetics (Medel et al. 2023; Brake et al. 2024), though we acknowledge that opposing results have been observed (Salvatore et al. 2024). Crucially, the E:I ratio is closely related to brain criticality (Beggs and Plenz 2003; Poil et al. 2012; Lombardi et al. 2017; Bruining et al. 2020; Ahmad et al. 2022; Liang et al. 2024; Javed et al. 2025) and is even discussed as the primary criticality control parameter (Fuscà et al. 2023), i.e., a variable that, when exceeding a critical value, induces a phase transition (O’Byrne and Jerbi 2022) or significant changes in the system’s state (Algom and Shriki 2026).

Empirically, increasing excitation over inhibition flattens the spectral slope, whereas increasing inhibition over excitation steepens it (Gao et al. 2017; Medel et al. 2023; Cortes-Briones et al. 2025). Simultaneously, an increase in excitatory over inhibitory neural activity causes a neural system to move toward a supercritical regime, while an increase in inhibition over excitatory neural activity causes a neural system to move toward a subcritical regime (Bruining et al. 2020; Javed et al. 2025; Algom and Shriki 2026). Thus, the spectral slope’s ability to reflect changes in the E:I ratio makes it a valuable proxy for brain criticality: steepening likely indicates a shift toward subcriticality, while flattening likely indicates a shift toward supercriticality. Similarly, LZC has been linked to brain criticality on both a theoretical (Carhart-Harris 2018) and experimental levels (Fekete et al. 2018; Toker et al. 2022; Maschke et al. 2024b; Pascarella et al. 2025; Mago et al. 2025). Consistent with the present results, evidence suggests that LZC may scale linearly with the branching parameter (Maschke et al. 2024b), potentially increasing from subcritical to supercritical states (von Wegner et al. 2024).

In the present study, steeper spectral slopes (higher values for mean PLE and mean spSlopeExp) and lower complexity (lower mean LZC values) at rest were associated with improved subsequent BCI performance in pALS. Since increased LZC values and flatter spectral slopes indicate a shift toward a supercritical regime, the correlations observed align with the assumption that BCI performance improves in pALS as their resting-state brain activity moves away from supercritical dynamics towards the critical transition point. These results replicate earlier findings with the pLIS-ALS of Dataset 1 (Settgast et al. 2023). Moreover, these results demonstrate that the pattern of a steeper spectral slope and lower complexity at rest relating to better BCI performance may generalize across pALS.

Alternatively, the association between a steeper spectral slope at rest and subsequent better BCI performance could also be interpreted in the context of recent findings in healthy participants. These studies have linked steeper spectral slopes at rest to better task performance (Immink et al. 2021; Thuwal et al. 2021; Cross et al. 2022), faster cognitive processing (Ouyang et al. 2020), higher cognitive ability (Pei et al. 2023), cognitive control (Pi et al. 2024), and general cognitive functioning (Euler et al. 2024). However, previous findings regarding spectral slopes at rest and subsequent BCI performance in healthy participants contradict this explanation (Settgast and Kübler 2024). In this study, flatter spectral slopes at rest were associated with better subsequent BCI performance. This indicates that the observed association between the steepening of the spectral slope in resting-state EEG and increased BCI performance in the pALS of the present study – probably – cannot be explained by the patterns observed between better cognitive processing and steeper spectral slopes in healthy participants. Instead, together with the results for healthy participants in the present study they indicate a criticality-based interpretation proposed by the *B-ARC framework* (Fig. 1).

The pattern observed for pALS contrasts sharply with our findings for healthy participants, where we observed an opposite relationship (Fig. 8): a positive correlation between the exponent of the avalanche size distribution (α) at rest and subsequent BCI performance (see Fig. 5 and Table 3). This suggests that a flatter distribution at rest – implying an increased probability of large neuronal avalanches – is associated with better subsequent BCI performance.

We interpret this pattern as an indication that BCI performance in healthy participants improves as preceding resting-state brain activity approaches criticality from a subcritical state. This interpretation aligns with the increasing probability of large neuronal avalanches when brain activity approaches and surpasses the critical point (e.g., Fekete et al. (2018)), as well as with studies associating enhanced cognitive functioning with the proximity to criticality at rest (Irrmischer et al. 2018; Mahjoory et al. 2019; Ezaki et al. 2020; Herzog et al. 2021; Xu et al. 2022; Müller et al. 2025; Xin et al. 2025; Harel et al. 2025; Yellin et al. 2025). Therefore, it is likely that the subsequent BCI performance of the healthy participants improved as their resting-state brain activity approached the critical point. This finding is further supported by the strong positive correlation for the avalanche duration distribution (τ), suggesting that an increased probability of longer-lasting neuronal avalanches at rest is associated with improved subsequent BCI performance.

These results replicate previous findings. Samek et al. (2016) reported that increasing long-range temporal correlations (LRTC) – a marker of approaching criticality (Algom and Shriki 2026) – during training improved subsequent motor imagery BCI performance. Furthermore, we recently observed that flatter spectral slopes (PLE) and increasing complexity (LZC) at rest were associated with better BCI performance in healthy participants (Settgast and Kübler 2024). Although the specific variables differ between these studies, it appears that, for healthy participants, markers indicating a shift toward criticality during preceding recordings are consistently associated with better subsequent BCI performance.

However, the generality of this relationship was recently challenged. Thielen (2025) found a positive, albeit non-significant, correlation between the steepness of the spectral slope at Oz – referred to as *lambda* – and performance with a code-modulated visual evoked potential (c-VEP) BCI in healthy participants. This implies that a steeper slope – i.e., dynamics further from criticality – was associated with better performance. This contrasts with the findings in Settgast and Kübler (2024) and challenges the hypothesis that proximity to criticality is positively associated with BCI performance. The weight of this contradiction is emphasized by the fact that the spectral slope supposedly changes globally similar across the scalp (Ouyang et al. 2020; Hill et al. 2022; Pi et al. 2024; Hebron et al. 2025). This suggests that the single electrode used in Thielen (2025) may have reflected the same scalp-wide change of the spectral slope shown in Settgast and Kübler (2024). However, this discrepancy may be paradigm-specific, meaning this result may be restricted to c-VEP BCIs. Until replicated and shown to be statistically robust across the scalp, the significant correlations across paradigms in the present study and in past studies (Samek et al. 2016; Settgast and Kübler 2024) strongly suggest that a shift toward criticality is associated with better subsequent BCI performance in healthy participants.

Taken together, these findings revealed a pattern of inverted relationships between resting-state brain criticality and subsequent BCI performance for healthy participants versus pALS (Fig. 8). Considering research on the pathophysiology of ALS, we propose that this apparent conflict can be resolved. Evidence suggests that the brain’s proximity to criticality changes in ALS. Not only do pALS exhibit an altered spatiotemporal organization of neuronal avalanches (Polverino et al. 2022, 2024), but it has also been repeatedly observed that pALS exhibit increasing connectivity (Iyer et al. 2015; Shahriari et al. 2015; Nasseroleslami et al. 2019; Govaarts et al. 2022) and synchronicity – up to a state of hyper-connectedness (Sorrentino et al. 2018). This is likely the result of a progressive loss of inhibitory brain activity (Turner and Kiernan 2012), which leads to hyperexcitability (Van den Bos et al. 2018; Gunes et al. 2022). These changes in the excitation-to-inhibition ratio (E:I) (Gunes et al. 2022) – recently corroborated by findings of flatter spectral slopes in resting-state MEG (Trubshaw et al. 2024) – not only push networks toward increased synchronization (Sorrentino et al. 2018), but likely also toward slightly supercritical dynamics (Heiney et al. 2021).

Simultaneously, sensory deprivation resulting from motor function impairment may further shift brain activity toward a supercritical regime. Because action and perception are tightly coupled (Rolfs and Schweitzer 2022), a progressive reduction in motor function may lead to sensory deprivation, altering the statistics of incoming sensory evidence and weakening the stabilizing influence of external inputs on neural dynamics (Churchland et al. 2010). The resulting reduction in structured afferent drive may engage maladaptive homeostatic mechanisms that perturb the interplay of excitation and inhibition (Chen et al. 2022), thereby biasing the system towards supercritical dynamics (Algom and Shriki 2026). Computational modeling supports this interpretation. In an entropy-maximizing recurrent network model of early auditory processing, Dotan and Shriki (2021) demonstrated that attenuated sensory input can destabilize networks optimized for efficient information processing, leading to the spontaneous emergence of hallucination-like activity and a transition to supercritical pattern formation dynamics, as observed in tinnitus. By analogy, prolonged motor impairment and the accompanying impoverishment of sensorimotor feedback in LIS-ALS may promote compensatory increases in recurrent activity, shifting cortical dynamics toward criticality or even into a supercritical regime.

The *B-ARC* (BCI-Aptitude through Resting-State Criticality) *framework* integrates these findings by proposing that the opposing correlation patterns reflect a shared mechanism: both groups (pALS and healthy participants) perform better the closer their resting-state brain activity is to the critical point (Fig. 1), yet they originate from different starting points. Healthy participants likely reside in a slightly subcritical regime, where increasing criticality markers (e.g., a less negative α value) reflect an approach to the critical optimum. Conversely, pALS likely reside in a slightly supercritical regime, where decreasing criticality markers (e.g., a more negative α value) reflect a reduction of supercritical brain activity and a shift towards the critical optimum. In short, we propose that irrespective of the starting point, approaching the critical optimum in resting-state EEG increases subsequent BCI performance. All roads lead to Rome.

Although the conducted group comparisons of the featured resting-state neurophysiological markers of criticality did not reach statistical significance – likely due to sample size limitations – most of the trends revealed by effect size analyses underpin the assumed supercritical brain dynamics in ALS (see Fig. 8). For example, the observed large effect for the avalanche duration distribution (τ) suggests a steeper distribution for healthy participants as compared to pALS. Moderate effect sizes suggesting higher neural branching parameter (σ) in pALS compared to healthy participants, and steeper avalanche size distributions (α) lend further support. Moreover, the putatively supercritical brain activity of pALS is supported by the inverted U-shaped pattern of correlations found for healthy participants versus pALS, which aligns with theoretical implications of the critical brain hypothesis (O’Byrne and Jerbi 2022; Müller et al. 2025). Furthermore, findings indicating hyperexcitable brain activity in ALS lend further evidence to this hypothesis. Finally, supercritical brain activity has been observed for other neural disorders characterized by excitation-dominated brain activity such as Alzheimer’s dementia (Javed et al. 2025) and stroke (Wu et al. 2025).

However, given the absence of statistically significant group differences, and our reluctance to interpret raw values – instead of relative changes – of the neuronal avalanche dynamics due to potential subsampling effects (Levina and Priesemann 2017), we must consider an alternative explanation. It remains theoretically possible that both pALS and healthy participants reside within the subcritical regime, approaching a performance optimum that lies below the critical point. In this scenario, the groups would approach this optimum from either a more (healthy participants) or less (pALS) subcritical state. Operating within a slightly subcritical regime indeed offers advantages: it likely retains the benefits of criticality while reducing the risk of runaway excitation when exceeding the critical threshold due to small perturbations at the point of criticality (Algom and Shriki 2026). Nonetheless, this interpretation would need to explain why surpassing or deviating from a point that is – in theory – optimal for information processing (O’Byrne and Jerbi 2022; Müller et al. 2025; Algom and Shriki 2026) in favor for a less optimal point, results in better BCI performance for pALS. Furthermore, our findings regarding absolute delta power potentially contradict this alternative account.

If resting-state brain dynamics closer to criticality facilitated better BCI performance in pALS, the observed increase in absolute delta power (see Fig. 2 and Fig. 4) could be an indicator of critical slowing-down – the tendency of a system close to criticality to demonstrate slower time scales and to take more time to relax after perturbation (Yellin et al. 2025; Algom and Shriki 2026). Notably, compared to the otherwise inverted correlations observed for healthy participants (see Fig. 8 for a summary), absolute delta power exhibited a similarly positive correlation with BCI performance in both groups. Furthermore, absolute delta power exhibited consistent, strong positive correlations with the offset of the aperiodic (1/f) component of the EEG power spectrum (spSlopeOffs_mean) across datasets (see Fig. 2 and Fig. 5, as well as the Online Resource 1, Figure S1), and this offset was positively correlated with subsequent BCI performance in healthy participants (Fig. 5). Accordingly, the offset of the aperiodic (1/f) component likely captured critical slowing-down in healthy participants, a view supported by the difficulty in disentangling true low-frequency oscillations (delta and theta) from the aperiodic component (Gerster et al. 2022, 2025). Accordingly, consistent with the B-ARC framework, the findings suggest that both pALS and healthy participants exhibited critical slowing-down (i.e., approaching the point of criticality), which in turn improved BCI performance. Importantly, unlike other criticality markers that seemingly scale monotonically from sub- to supercritical regimes (like α, τ, or σ), critical slowing-down is expected to peak at criticality (Yellin et al. 2025).

However, our findings contrast with Shin et al. (2020), who found a negative correlation between frontal delta power at rest and subsequent BCI performance in healthy participants. This discrepancy may stem from important methodological differences: Shin et al. (2020) used eyes-closed resting-state recordings and their findings were restricted to frontal electrodes, whereas our study employed eyes-open resting-state recordings and delta power was averaged across all electrodes. Borgheai et al. (2024) reported another negative association between delta-frequency band power and BCI performance. Despite the small effect size (*β* = − .01), this negative regression coefficient implies a negative association with subsequent BCI performance, contradicting our reported results. Interestingly, the models in Borgheai et al. (2024) were trained on pooled data from healthy participants and pALS, suggesting a group-independent factor for this specific BCI protocol. However, this effect was only found for one of three protocols and was restricted to a subset of electrodes. Therefore, future empirical replication is required to determine whether these discrepancies are due to methodological differences (e.g., eyes open vs. closed, scalp-wide calculation vs. regions of interest), the specifics of the BCI paradigms (e.g., P300-based vs. visuo-mental BCI protocols) or due to our limited sample size as compared to Shin et al. (2020).

Notably, two of the criticality markers investigated here have recently been shown to exhibit associations with BCI use (respectively an EOG-based communication system in (Adama and Bogdan 2025)) in pCLIS. Bettencourt and Nunes (2024) reported steeper spectral slopes in the EEG of a person in CLIS during sessions with good BCI performance (> 70% accuracy) compared to sessions with low performance. However, a direct comparison to what we have reported here is difficult as they obtained their values during the BCI use. Likewise, LZC values have been shown to be indirectly related to performance with an EOG-based communication system in a person in CLIS. Specifically, Adama and Bogdan (2025) developed an approach to infer consciousness in pCLIS based on EEG data referred to as *normalized consciousness levels*. This inference was amongst others largely driven by LZC values and showed correlations with the performance achieved with the communication system in one of their pCLIS.

A recent study reported findings in pCLIS that seemingly contradict our supercriticality assumption for ALS. Zilio et al. (2023) found steeper spectral slopes (higher PLE values) and lower complexity (lower LZC values) in pCLIS as compared to healthy participants. They also observed high intra-individual fluctuations, inter-subject variability, and increasingly lower LZC and higher PLE values the longer the people were in CLIS. The authors interpreted these results as indicators for altered arousal in these people. However, this contrasts with the flattened spectral slope for non-CLIS pALS suggested by our effect size trends and reported in the literature (Trubshaw et al. 2024). This apparent contradiction might be resolved by the disease progression mechanism proposed by Clark et al. (2015). Reviewing the dysfunction of inhibitory regulation in an ALS mouse model, they propose a transition from hyperexcitability to hypoexcitability during the course of the motor neuron decline in ALS. Our sample potentially captured the hyperexcitable, supercritical phase where a steeper slope at rest was beneficial for subsequent BCI performance. In contrast, the pCLIS investigated by Zilio et al. (2023) may have represented an ever increasingly hypoexcitable, subcritical end phase of the disease where a flatter slope at rest might be beneficial to support conscious awareness. However, whether these mouse model results (Clark et al. 2015) translate to humans, and whether a flattening of the spectral slope in CLIS may indicate WoC remains an open empirical question.

If a flattening of the spectral slope is indeed related to better BCI performance in pCLIS, this pattern would readily be integrated into the B-ARC framework. Within this framework, the mechanism linking the proximity to criticality to BCI performance is what matters, regardless of whether the starting point is in a slightly sub- or supercritical regime.

Lastly, instead of waiting for WoC to naturally occur, real-time criticality tracking could offer the opportunity to alter the proximity to criticality via targeted interventions. For example, Gervais et al. (2023) proposed using psychedelics to modulate subcritical states in DOC. Indeed, recently Cardone et al. (2025) investigated the effect of the atypical psychedelic ketamine in people with prolonged DOC, finding that LZC increased after infusion along with increased arousal. Another approach was proposed in Polyakov et al. (2025). They modeled how in-silico brain stimulation can be tailored to induce healthy-like EEG power spectra in models fitted to people with DOC. These initial results imply that real-time adapted interventions on brain criticality may eventually support opening WoC.

### Limitations

The primary limitation of this study is its small sample size. Although we used effect sizes to identify potential trends, these findings require replication in larger cohorts. Therefore, these results should be considered preliminary until replicated, even though they are conceptually consistent with previous findings (Settgast and Kübler 2024) and the literature indicating optimal information processing capacity at the point of criticality (Müller et al. 2025) and putatively supercritical dynamics in ALS due to ALS-related hyperexcitability (Gunes et al. 2022) and the association between excessive excitation and supercritical dynamics (e.g., (Javed et al. 2025)). Due to the absence of repeated measures for healthy participants, we had to rely on between-subject correlations. Therefore, we cannot rule out Simpson’s paradox (Bakdash and Marusich 2017), where individual within-subject associations potentially diverge from the observed between-subject pattern. If such a paradox were present in the data, the seemingly opposing patterns for pALS and healthy participants might vanish. A further methodological limitation concerns subsampling. Neuronal avalanche criticality dynamics can be biased by the number of electrodes (Levina and Priesemann 2017). Consequently, we refrained from interpreting raw values and focused instead on relative changes and correlations. We further acknowledge that we reduced the number of observations in the rmCorr analysis of the pALS in Dataset 2 due to the quality control procedure. Although this rigorous strategy resulted in the loss of data points, it also ensured that the criticality markers were not calculated using artifact-prone data, thus decreasing the probability that reported effects are artifacts only. Along with rejecting entire sessions, however, this strategy has also led to the condition that not every criticality marker was calculated using the same length of data. We also acknowledge that we have treated repeated observations as independent to visualize the inter-correlations between the resting-state neurophysiological markers of criticality for the pALS of Dataset 2. We strongly emphasize that we did not use these correlations to answer our actual research question – i.e., the association between resting-state brain dynamics and subsequent BCI performance – but significance levels for these statistics might have been inflated (Bland and Altman 1994). Finally, consistent with the exploratory nature of this study we decided to not correct for multiple comparisons (Bender and Lange 2001). Nonetheless, a major strength of this study is the replication of findings across two independently recorded datasets (Szucs and Ioannidis 2017). The consistent within-subjects correlations observed in the single person in the locked-in state after a disease history with ALS (Dataset 1) and the group of 5 pALS (Dataset 2) provide encouraging evidence that these patterns may be robust within this target population.

## Conclusion

In this exploratory study, we investigated the correlations between resting-state brain dynamics in EEG, as quantified via neurophysiological criticality markers, and subsequent brain-computer interface (BCI) performance in healthy participants and people with ALS. Across two independent datasets, people with ALS exhibited consistent correlations between BCI performance and neuronal avalanche dynamics, absolute delta power, the exponent of the aperiodic (1/f) component of the EEG power spectrum, and Lempel-Ziv complexity, all of which were derived from preceding resting-state EEG. In contrast, a small group of healthy participants showed an inverted correlation pattern.

Building on the critical brain hypothesis, we propose the *B-ARC* (BCI-Aptitude through Resting-State Criticality) framework to reconcile these findings. Within this brain-state dependent framework, BCI performance improves as resting-state brain activity approaches the critical point: Healthy participants approach this point from a slightly subcritical regime, whereas people with ALS appear to approach it from a putatively supercritical regime.

Although the group comparisons of resting-state neurophysiological markers of criticality did not reach statistical significance – likely due to small sample size limitations –, the large and medium effect sizes and consistency with the observed hyperexcitability in ALS suggest supercritical dynamics for these people, providing a coherent interpretational framework to explain these otherwise contradictory correlation patterns.

This study provides the first evidence of consistent correlations between resting-state brain dynamics in EEG – captured via neurophysiological markers of criticality – and subsequent BCI performance in people with ALS across datasets. By integrating these findings with data from healthy participants, we propose the *B-ARC framework*, which indicates that BCI aptitude relies on the proximity to criticality at rest. If the observed correlates can be further replicated and if they generalize to people in the complete locked-in state, they may help to identify Windows of Consciousness (Kübler 2020) in the future, potentially providing a puzzle piece to unlock the complete locked-in state.

## Supplementary Material

Supplementary Files

This is a list of supplementary files associated with this preprint. Click to download.

- OnlineResource1.pdf
- OnlineResource2.txt
- OnlineResource3.txt
- OnlineResource4.txt

## Figures and Tables

**Figure 1:**
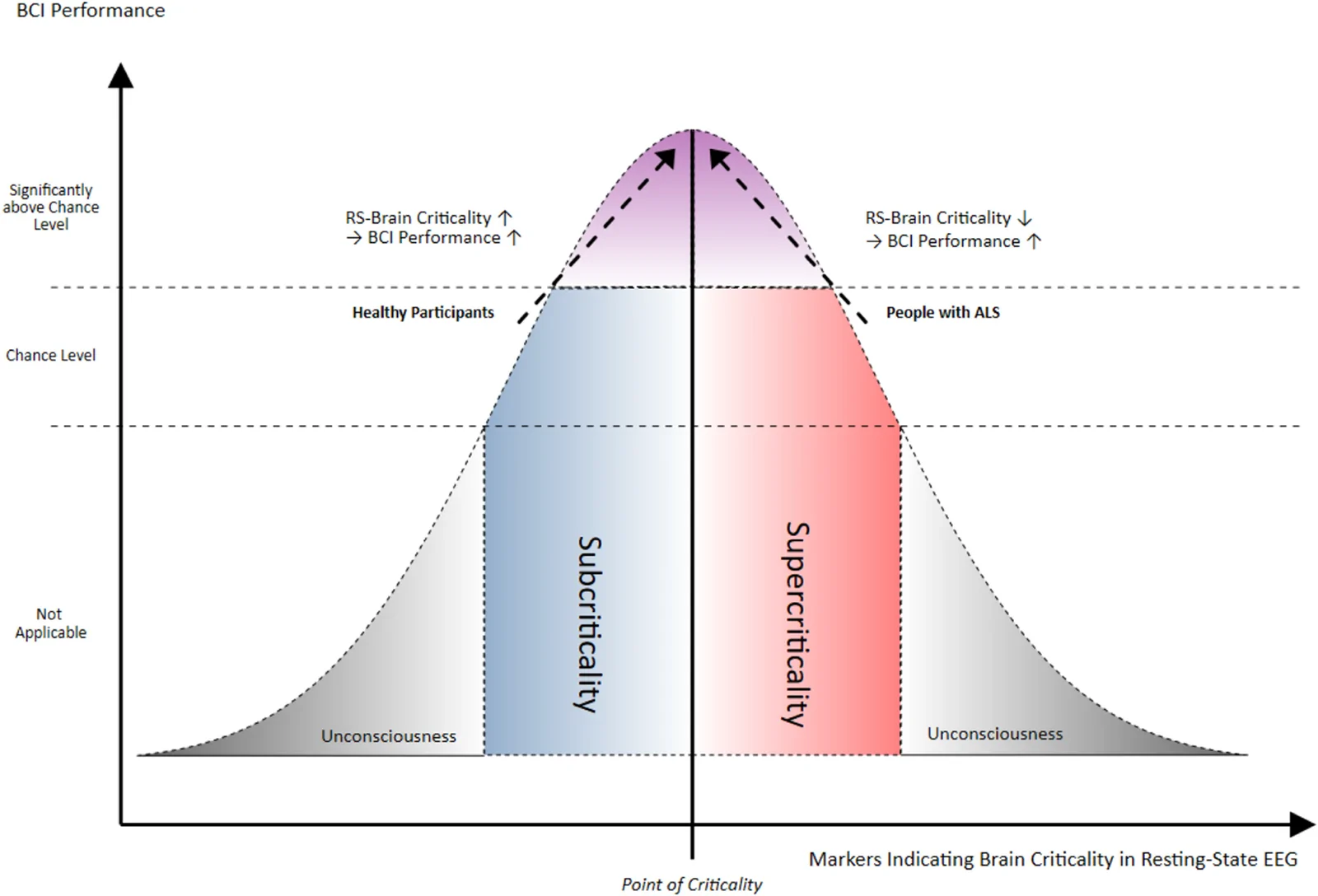
The B-ARC (BCI-Aptitude through Resting-State Criticality) framework posits a non-linear, inverted U-shaped relationship between resting-state brain criticality and subsequent BCI performance. According to B-ARC, optimal performance is achieved near the point of criticality. Both healthy participants and people with ALS (pALS) improve BCI performance as their resting-state brain activity approaches this critical optimum, albeit from opposite directions. pALS move toward criticality from a putatively supercritical regime of brain activity, whereas healthy participants approach criticality from a slightly subcritical state

**Figure 2:**
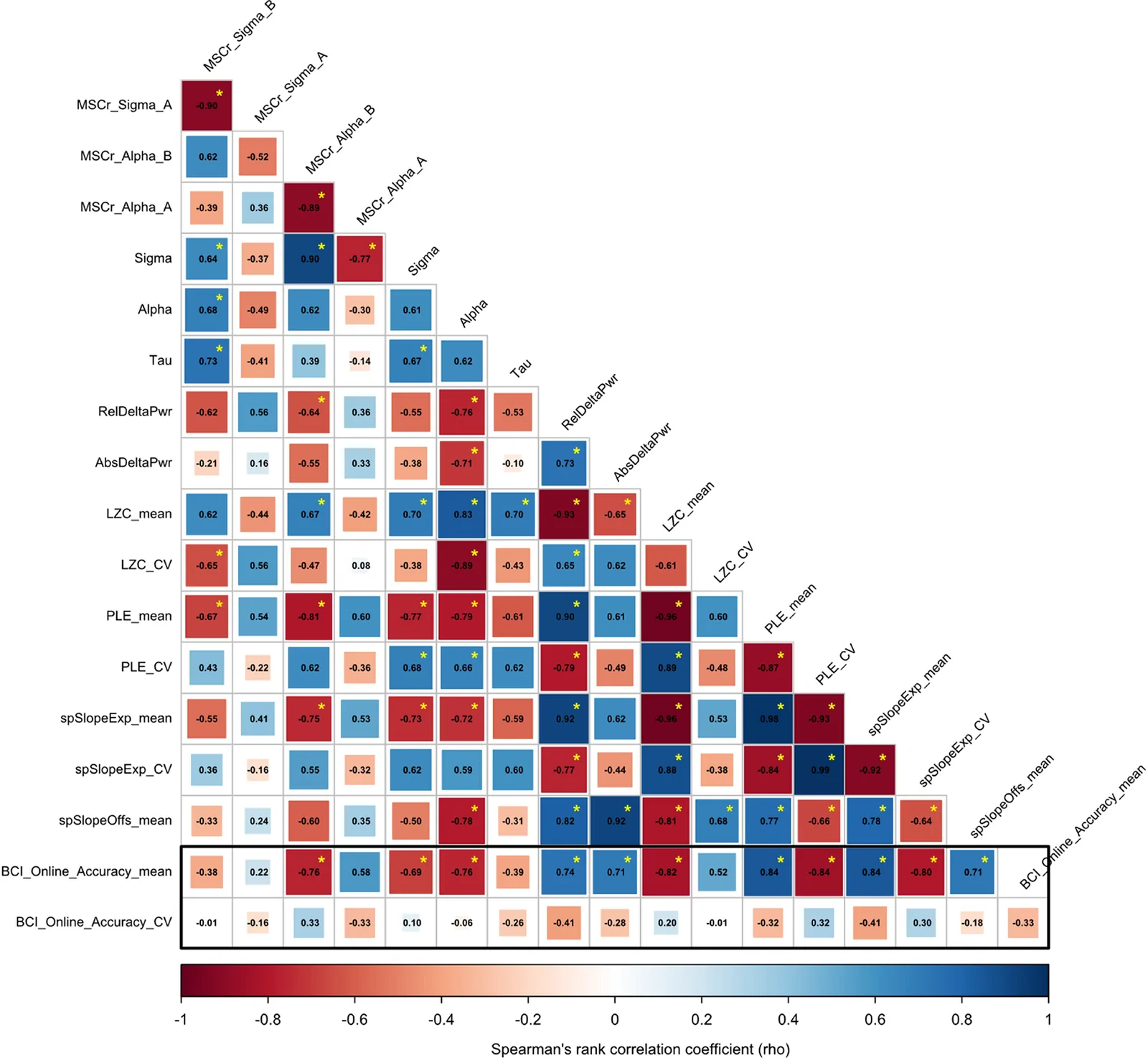
The correlogram shows Spearman’s rank correlation coefficients (ρ; rho) between criticality markers derived from resting-state EEG and subsequent BCI performance metrics (BCI_Online_Accuracy_mean and BCI_Online_Accuracy_CV) for dataset 1. Asterisks indicate uncorrected significance at p < .05. Test statistics for significant correlations with mean BCI performance (BCI_Online_Accuracy_mean) are provided in Table 2. Abbreviations: MSCr_Sigma/Alpha_A/B: slope/intercept of σ/α as a function of timescales; Rel/AbsDeltaPwr: relative/absolute delta power; LZC: Lempel-Ziv complexity; PLE: power-law exponent; spSlopeExp/Offs: exponent/offset of the aperiodic (1/f) component of the EEG power spectrum (FOOOF); CV: coefficient of variation

**Figure 3:**
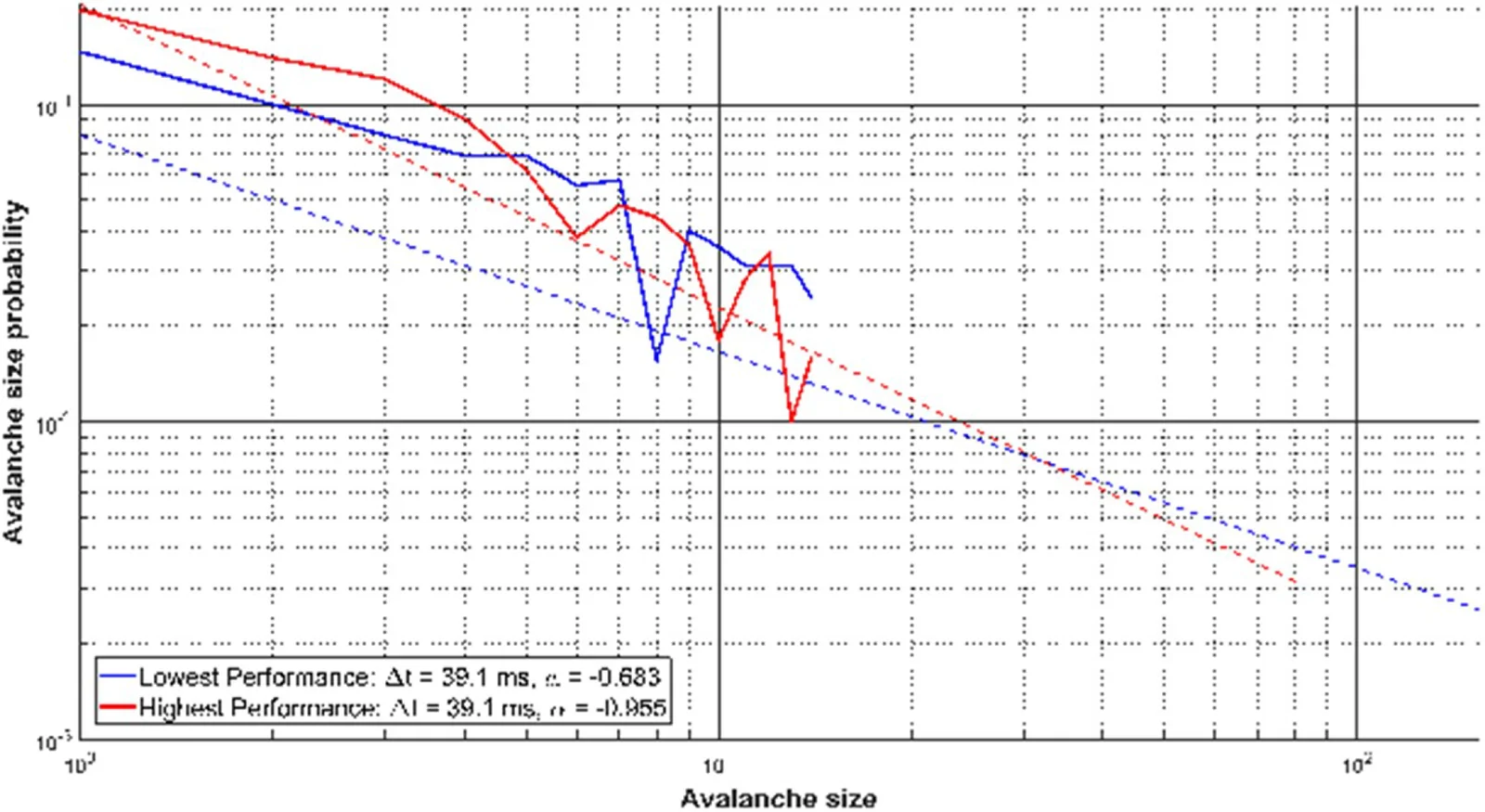
The plot illustrates the distributions for the sessions corresponding to the lowest (50%; blue) and highest (83%; red) mean BCI performance for dataset 1. The solid lines represent the distributions of avalanche sizes in the EEG recording, and the dashed lines indicate their corresponding power law fits with the exponents (α) shown in the legend

**Figure 4:**
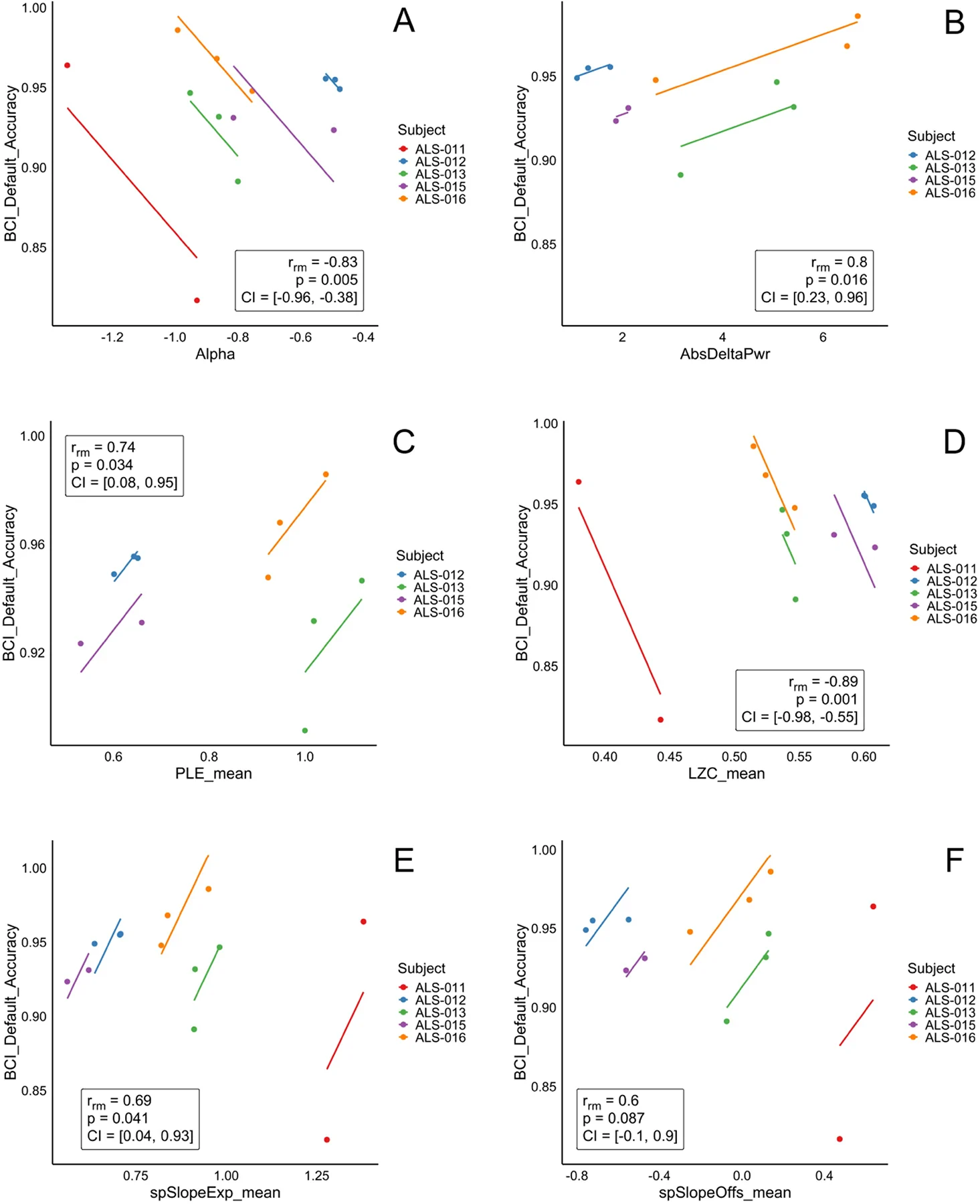
The plot shows the common within-individual associations (rmCorr) between BCI performance and 6 resting-state criticality markers for pALS in Dataset 2. Each panel provides the correlation coefficient (r_rm_), its 95% confidence interval (CI), and the exact p-value. A-F: The rmCorr between BCI performance (BCI_Default_Accuracy) and (A) the exponent of the avalanche size distribution (α, Alpha), (B) absolute delta power (AbsDeltaPwr), (C) mean power-law exponent (PLE_mean), (D) mean Lempel-Ziv complexity (LZC_mean), (E) mean exponent of the aperiodic (1/f) component of the EEG power spectrum (FOOOF) (spSlopeExp_mean), as well as (F) the mean offset of the aperiodic (1/f) component of the EEG power spectrum (FOOOF) (spSlopeOffs_mean). **Note:** Analyses for absolute delta power (B) and mean power-law exponent (C) were calculated after the exclusion of person ALS-11 due to influential outlier behavior (N =11 sessions from 4 pALS). All other panels (A, D, E, and F) represent rmCorr analyses for the full sample (N = 13 sessions from 5 pALS). For rmCorr results regarding (B) and (C) prior to exclusion, see Online Resource 1, Figure S3

**Figure 5:**
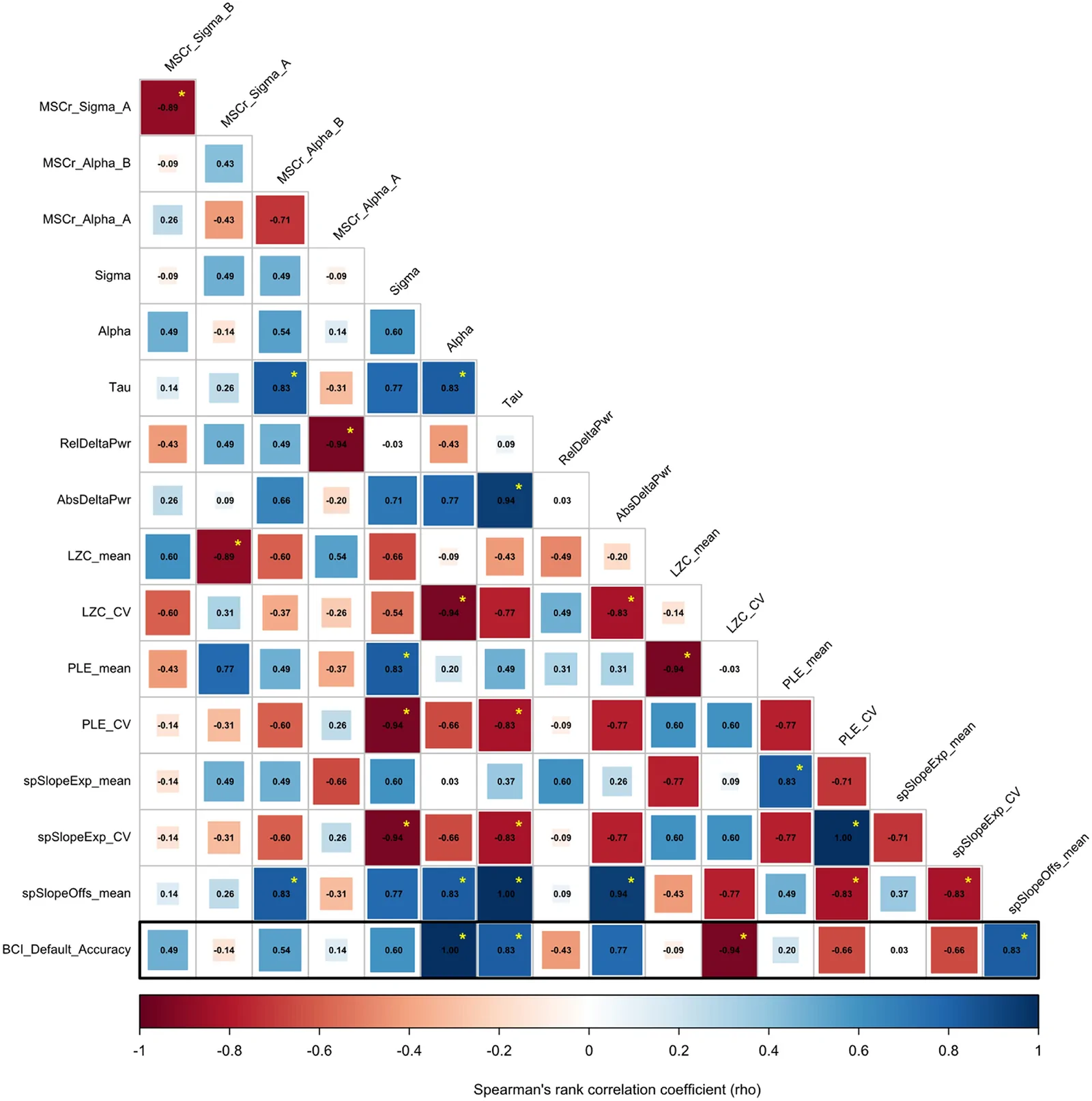
The correlogram displays the Spearman’s rank correlation coefficients (ρ; rho) between neurophysiological criticality markers derived from resting-state EEG and subsequent BCI performance (BCI_Default_Accuracy) for the healthy participants of dataset 2. Asterisks indicate uncorrected significance at p < .05. The test statistics for significant correlations with BCI performance are provided in Table 3

**Figure 6:**
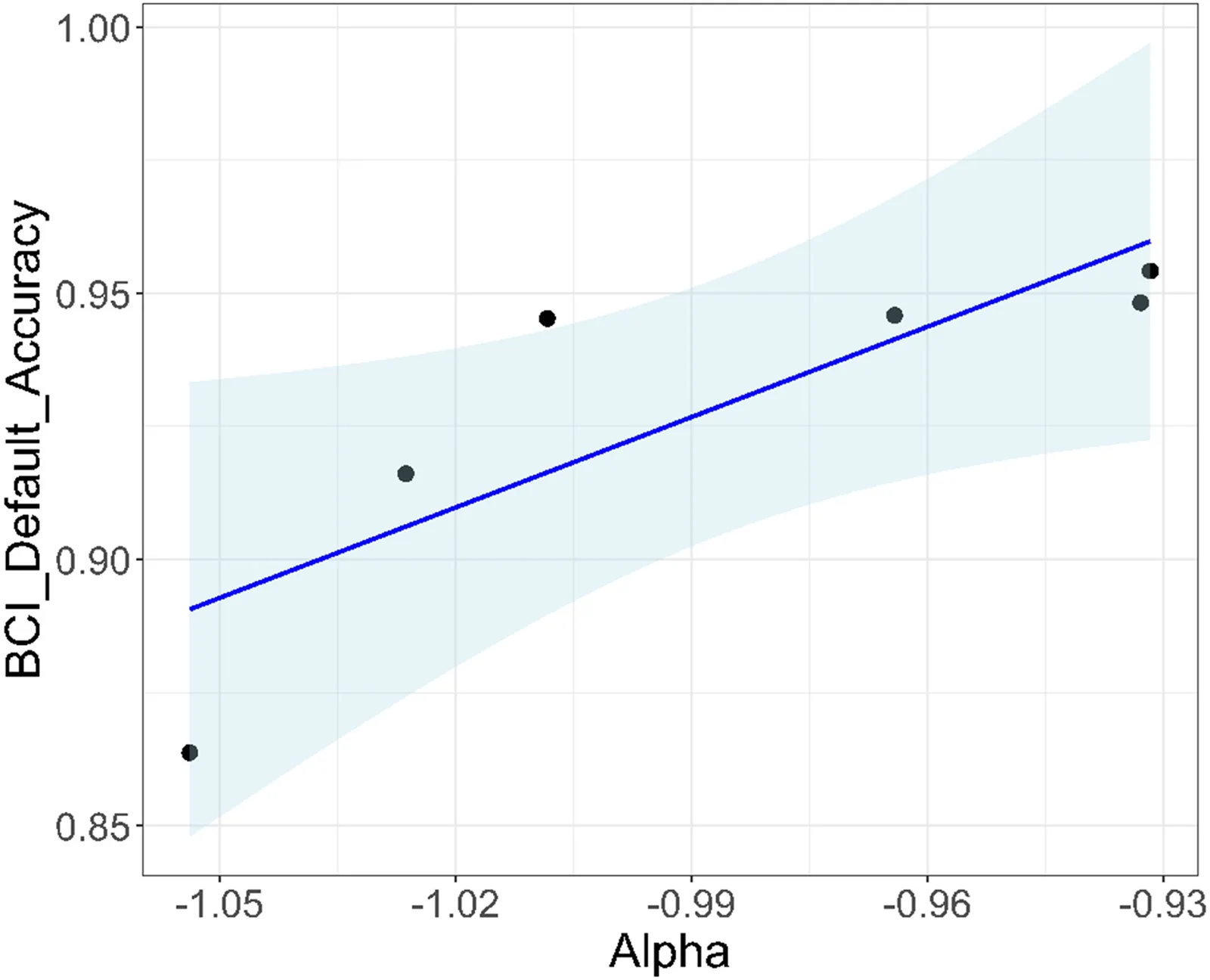
This plot illustrates the association between the avalanche size distribution (α) at rest and subsequent BCI performance (BCI_Default_Accuracy) for the healthy participants (N = 6) of Dataset 2. The solid blue line represents the linear regression fit, and the light blue shaded region indicates its 95% confidence interval

**Figure 7:**
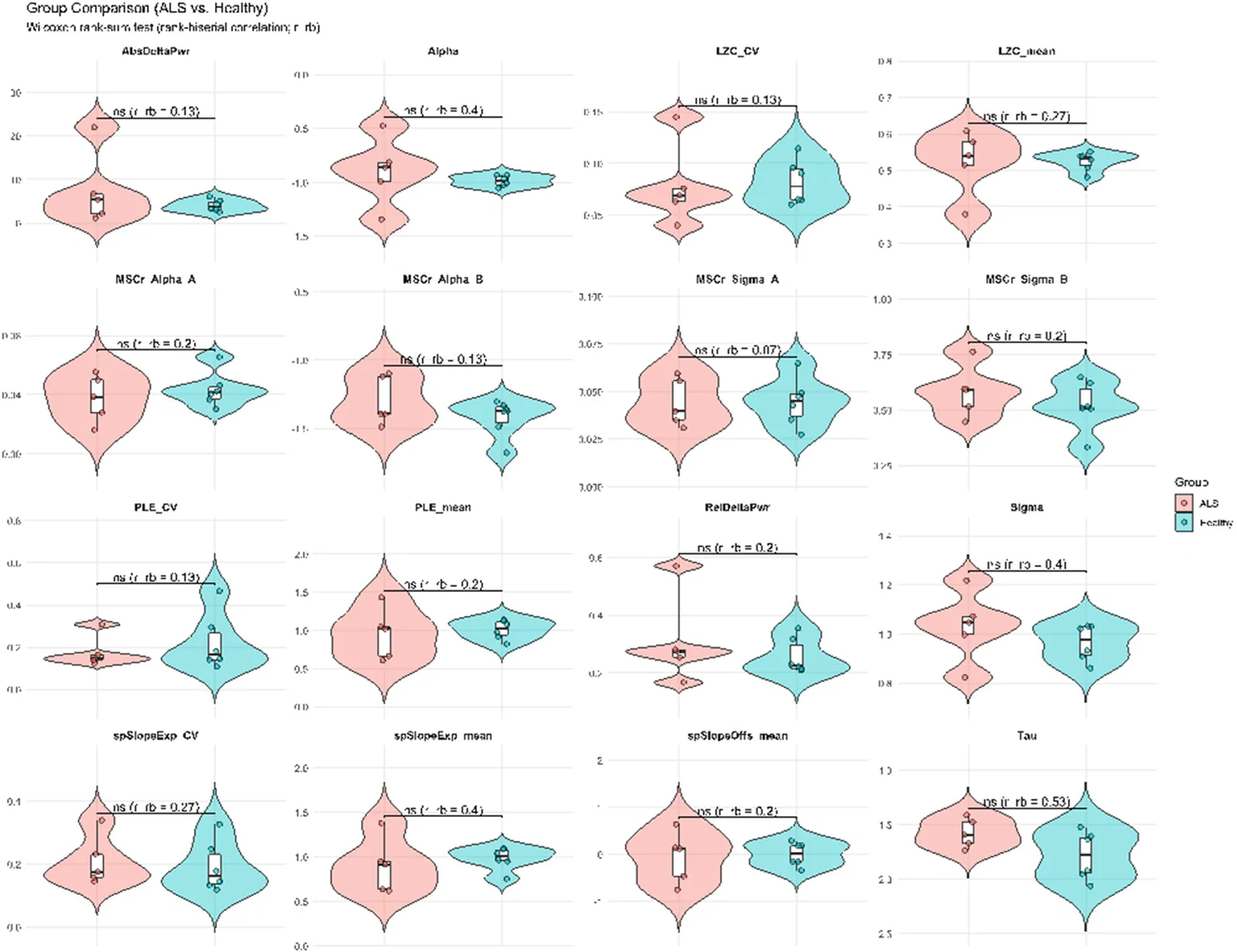
Violin plots illustrate the data distribution for each criticality marker for pALS and healthy participants of dataset 2. No comparison (Wilcoxon rank-sum tests) reached statistical significance. Detailed test statistics are provided in Table 4. Abbreviations: ns: non-significant; r_rb: rank-biserial correlation (effect size)

**Figure 8:**
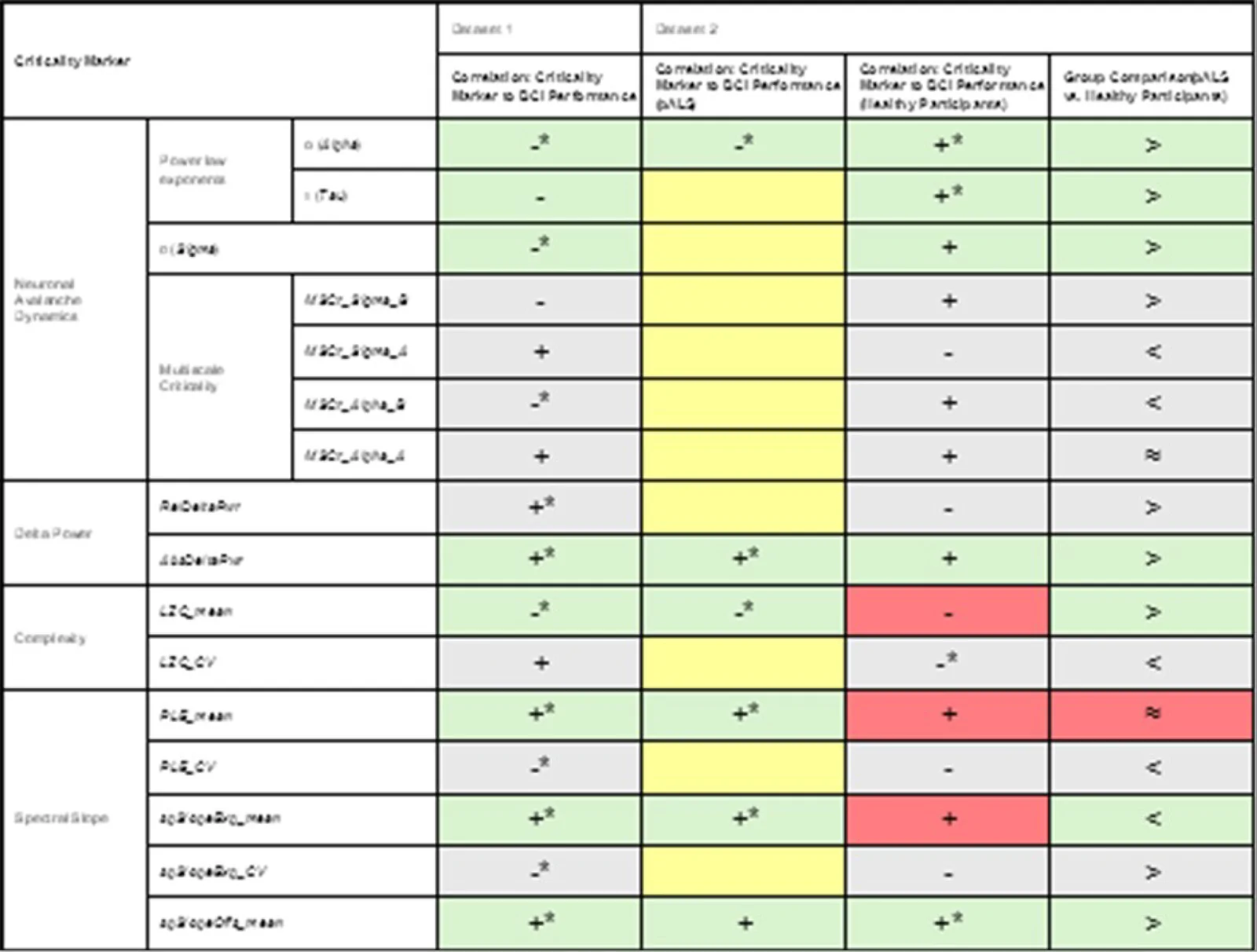
This overview summarizes correlations between each criticality marker at rest and subsequent BCI performance across datasets and groups. It also indicates trends for the group comparisons between the people with ALS (pALS) and healthy participants of Dataset 2. Symbols: [+] refers to a positive correlation, [−] refers to a negative correlation, [>] indicates a higher value for pALS, [<] indicates a higher value for healthy participants (irrespective of magnitude), [≈] indicates approximately similar values for pALS and healthy participants. Asterisks (*) indicate uncorrected significance at p < .05. Color Coding: light green cells align with the proposed B-ARC framework and the assumption for supercritical brain dynamics in ALS (Figure 1, see Discussion for details); light red cells indicate deviations; light yellow cells indicate that rmCorr prerequisites were not fulfilled (i.e., inconsistent directionality across subjects, as verified by spaghetti plots); light gray cells indicate an unclear relationship to the proposed framework. For a detailed description of individual results, see the preceding sections.

**Table 1:** Overview of the analyzed neurophysiological markers of criticality.

Criticality marker			Definition	Operationalization	Key parameter	Key reference
Neuronal avalanche dynamics	Power law exponents	α (*Alpha*)	Avalanche size distribution	Exponent of the power law fit to the avalanche size distribution	Timescale (Δt): 10 samples (~ 39.1 ms) Threshold: ± 2 SD	(Shriki et al. 2013)
		τ (*Tau*)	Avalanche duration distribution	Exponent of the power law fit to the avalanche duration distribution		
	σ (*Sigma*)		Branching parameter	Average ratio between the number of events in the second and first time bin of an avalanche		
	Multiscale criticality	*MSCr_Sigma_B*	Intercept ofσ as a function of timescales		Analysis timescales: 6–14 samples (in steps of 2)	(Fekete et al. 2021)
		*MSCr_Sigma_A*	Slope of σ as a function of timescales			
		*MSCr_Alpha_B*	Intercept of α a as a function of timescales			
		*MSCr_Alpha_A*	Slope of α as a function of timescales			
Delta power (approximation for assessing critical slowing-down, (Yellin et al. 2025))	*RelDeltaPwr*		Relative delta-frequency band power	Mean (across channels) absolute delta-frequency band power divided by the absolute power across the entire spectrum	Delta-frequency band: 1–4 Hz Entire spectrum: 1–40 Hz	
	*AbsDeltaPwr*		Absolute delta-frequency band power	Mean (across channels) delta-frequency band power	Delta-frequency band: 1–4 Hz	
Complexity	*LZC_mean*		Mean LZC	Mean of LZC (pattern richness) across time windows and channels		(Zilio et al. 2023)
	*LZC_CV*		Mean CV of LZC	Mean CV (across channels) for LZC across time windows		
Spectral slope	*PLE_mean*		Mean PLE	Mean PLE (absolute value of the slope of the linear least square regression for the log-log transformed PSD) across channels and time windows	Spectrum (PSD): 1–40 Hz	
	*PLE_CV*		Mean CV of the PLE	Mean (across channels) CV for PLE across time windows		
	*spSlopeExp_mean*		Mean spectral slope exponent	Mean (across channels and time windows) exponent of the aperiodic (1/f) component of the PSD using FOOOF	Spectrum (PSD): 1–40 Hz Fixed aperiodic mode (without knee) Minimum peak width: 4 Hz Maximum peak width: 8 Hz	(Donoghue et al. 2020b)
	*spSlopeExp_CV*		Mean CV of the spectral slope exponent	Mean (across channels) CV of the spectral slope exponent		
	*spSlopeOffs_mean*		Mean spectral slope offset	Mean (across channels and time windows) offset of the aperiodic (1/f) component of the PSD using FOOOF		

This table lists the neurophysiological markers of criticality that have been analyzed in resting-state EEG. It provides a short definition of each marker, an operationalization, key parameters for calculation, and a key reference. Variable names are indicated in italics. For details, please refer to the relevant subsection below. Abbreviations: LZC: Lempel-Ziv complexity; PLE: power-law exponent.

**Table 2:** Significant correlations between resting-state neurophysiological criticality markers and mean BCI performance (pLIS-ALS, Dataset 1)

Criticality Marker	Spearman’s ρ	95% confidence interval (Sperman’s ρ)	p-Value
MSCr_Alpha_B	− .76	[−.94, − .23]	.010
σ (Sigma)	− .69	[−.92, − .08]	.028
α (Alpha)	− .76	[−.94, − .22]	.011
RelDeltaPwr	.74	[.18, .94]	.015
AbsDeltaPwr	.71	[.12, .93]	.022
LZC_mean	− .82	[−.96, − .38]	.003
PLE_mean	.84	[.43, .96]	.002
PLE_CV	− .84	[−.96, − .43]	.002
spSlopeExp_mean	.84	[.43, .96]	.002
spSlopeExp_CV	− .80	[−.95, − .32]	.006
spSlopeOffs_mean	.71	[.13, .93]	.021

The table lists the Spearman’s rank correlation coefficients (ρ), uncorrected p-values, and 95% confidence intervals for all resting-state criticality markers that showed significant correlations with mean BCI performance. A complete list of all correlations is provided in Online Resource 1 (Table S1).

**Table 3:** Significant correlations between resting-state neurophysiological markers of criticality and BCI performance (healthy participants, Dataset 2)

Criticality Marker	Spearman’s ρ	95% confidence interval (Sperman’s ρ)	p-Value
α (Alpha)	1	[1 1]	< .001
τ (Tau)	.83	[.02, .98]	.042
LZC_CV	− .94	[−.99, − .54]	.005
spSlopeOffs_mean	.83	[.02, .98]	.042

The table lists the Spearman’s rank correlation coefficients (ρ), uncorrected p-values, and 95% confidence intervals for all resting-state neurophysiological criticality markers that showed significant correlations with BCI performance (BCI_Default_Accuracy). A complete list of all correlations is provided in Online Resource 1, Table S3.

**Table 4:** Group comparisons (Wilcoxon rank-sum tests) between pALS and healthy participants of Dataset 2 (criticality markers)

Variable	pALS: Median (IQR)	Healthy Participants: Median (IQR)	U-Value	p-Value	Effect Size (rank-biserial correlation; r_rb_)	Hodges-Lehmann estimate	95% CI (Hodges-Lehmann estimate)
AbsDeltaPwr	5.41 (4.57)	3.82 (1.76)	17	.792	.13	.92	[−3.23, 17.74]
α (Alpha)	−.86 (.18)	− .99 (.08)	21	.329	.4	.11	[−.34, .49]
LZC_CV	.07 (.01)	.08 (.03)	13	.792	.13	− .01	[−.05, .05]
LZC_mean	.54 (.06)	.53 (.03)	19	.537	.27	.02	[−.15, .08]
MSCr_Alpha_A	.04 (.02)	.04 (.01)	12	.662	.2	− .01	[−.03, .01]
MSCr_Alpha_B	−1.39 (.27)	−1.38 (.12)	17	.792	.13	.14	[−.11, .37]
MSCr_Sigma_A	.04 (.02)	.05 (.01)	14	.931	.07	0	[−.02, .02]
MSCr_Sigma_B	.59 (.08)	.51 (.09)	18	.662	.2	.08	[−.11, .26]
PLE_CV	.15 (.02)	.16 (.12)	13	.792	.13	− .01	[−.3, .13]
PLE_mean	1.02 (.38)	1.02 (.17)	12	.662	.2	− .09	[−.47, .36]
RelDeltaPwr	.27 (.03)	.23 (.08)	18	.662	.2	.04	[−.08, .34]
σ (Sigma)	1.05 (.07)	.98 (.11)	21	.329	.4	.06	[−.11, .21]
τ (Tau)	−1.59 (.19)	−1.78 (.33)	23	.177	.53	.21	[−.1, .51]
spSlopeExp_CV	.17 (.07)	.16 (.09)	19	.537	.27	.02	[−.1, .16]
spSlopeExp_mean	.91 (.31)	1 (.12)	9	.329	.4	− .13	[−.45, .34]
spSlopeOffs_mean	.12 (.61)	.01 (.33)	12	.662	.2	− .06	[−.75, .49]

The table presents descriptive statistics (median and IQR), the test statistics for the Wilcoxon rank-sum tests (U-, and p-value), their effect sizes (rank-biserial correlation), the Hodges-Lehmann estimates, and their 95% confidence intervals for the group comparison between pALS and healthy participants of Dataset 2 for all resting-state neurophysiological markers of criticality.

## Data Availability

Data from Dataset 1 are available from the corresponding author of the original study (Ziebell et al. 2024) upon reasonable request. Data from Dataset 2 are available from the corresponding author of the original study (Borgheai et al. 2024) upon reasonable request.

## Footnotes

1. For a fNIRS-based BCI, see Borgheai et al. (2020) who demonstrated consistent accuracies in a person diagnosed with ALS and without reliable means of communication.

2. In this paper, *spectral slope* is used as an umbrella term. The cited literature refers to this EEG power spectrum component using various terms, including *1/f*, *aperiodic*, *non-periodic*, or *scale-free activity*, *1/f slope*, *1/f* or *aperiodic scaling*, *spectral*, or *power-law exponent*, *power law distribution*, or *lambda*.

3. For example, see Brake et al. (2024), who investigated the neurophysiological origin of the spectral slope by systematically changing the proximity to criticality in their model. For more theoretical work linking avalanche power laws to power spectra, see Apicella, et al. (2022), Lombardi et al. (2017, 2019) and Nandi et al. (2022). Also, see the repeatedly made association between changes in the spectral slope and brain criticality in experimental studies (Ouyang et al. 2020; Thuwal et al. 2021; Wainio-Theberge et al. 2022; Pei et al. 2023; Maschke et al. 2024b; Pascarella et al. 2025; Tucker et al. 2025), as well as Hengen and Shew (2025), who referred to the 1/f power spectrum as an analytical approach to criticality in a recent review.

## Data Availability

Data from Dataset 1 are available from the corresponding author of the original study [DOI: 10.31234/osf.io/abjyw] upon reasonable request. Data from Dataset 2 are available from the corresponding author of the original study [DOI: 10.1016/j.compbiomed.2023.107658] upon reasonable request.
